# Exploring functional InDels and genetic diversity: agro-morphometric and molecular insights into the Western and Eastern gene pools of carrot (*Daucus carota* L.)

**DOI:** 10.3389/fpls.2025.1658653

**Published:** 2025-10-22

**Authors:** Trupthi Mudihal, Sarvamangala Cholin, Chaitra C. Kulkarni, K. M. Shivaprasad, Prakash Kumar, William Rolling, Philipp Simon

**Affiliations:** ^1^ Plant Molecular Biology Lab (DBT-BioCARe), Department of Biotechnology & Crop Improvement, College of Horticulture, Bagalkot, University of Horticultural Sciences, Bagalkot, Karnataka, India; ^2^ Kittur Rani Channamma College of Horticulture, Arabhavi, University of Horticultural Sciences, Bagalkot, Karnataka, India; ^3^ Indian Council of Forestry Research and Education (ICFRE)-Institute of Forest Biodiversity, Hyderabad, India; ^4^ ICAR-Indian Agricultural Statistics Research Institute, New Delhi, India; ^5^ Department of Plant & Agroecosystem Sciences, University of Wisconsin, Madison, WI, United States; ^6^ USDA-ARS Vegetable Crops Research Service, Madison, WI, United States

**Keywords:** carrot, gene pool, InDels, agro-morphometric, genetic diversity, coding region, population structure

## Abstract

Carrot (*Daucus carota* L.) is a globally cultivated root vegetable with significant genetic diversity. This first study generated and validated carrot InDels to unravel the genetic divergence between Eastern and Western gene pools, integrating agro-morphometric traits with functional InDel markers. Eastern accessions exhibited larger plants, bigger roots with diverse colors, while Western accessions were more uniform orange color and compact in architecture. From RNA-seq data, 271 agarose-resolvable functional InDels (>15bp length difference) were identified, of which 48 validated markers showed high polymorphism (84.21%) across two gene pools supporting secondary domestication changes. Located in coding and UTR regions, these InDels likely regulate gene expression and may have contributed to significant genetic modifications among carrot gene pools. Genetic diversity in the Western gene pool indicated more intense selection and domestication. Population structure and phylogenetic analysis revealed clear gene pool differentiation (Fst = 0.181) with potential gene flow (Nm = 1.716). Functional annotation of linked InDels to key biological processes, highlighted their role in domestication. Key InDels (DcFInDel32, DcFInDel28, and DcFInDel55) were associated with multiple traits, underscoring their utility in marker-assisted selection (MAS). These findings provide insights for developing improved carrot cultivars with high yield and quality adapted to diverse climates.

## Introduction

Carrot (*Daucus carota* subsp. *sativus* L.) is an ancient, cool-season root vegetable of the Apiaceae family with a diploid genome of 473 Mb and 2n = 2x = 18 (Iorizzo et al., 2016). Native to Afghanistan region, carrots are now cultivated globally for their rich nutritional profile, which includes high levels of carotenoids, antioxidants, vitamins, and minerals, contributing to their well-known immune-boosting, anti-inflammatory, and antioxidant properties (Kulkarni et al., 2023). Originally, carrots were valued more for their flavorful leaves and seeds than their roots. Over time, however, selective breeding has led to the domestication of the root, producing varieties with orange, purple, white, yellow, and red, with the orange type being commercially dominant (Bradeen and Simon, 2007).

Carrot germplasm exhibits considerable phenotypic diversity, with cultivated varieties broadly classified into Eastern and Western types based on morphological and evolutionary characteristics (Coe et al., 2023; Cholin et al., 2024). The Eastern varieties, often referred to as Asiatic carrots (*Daucus carota* ssp. *sativus* var. *atrorubens* Alef.), were domesticated over 1,100 years ago in Central and Eastern Asia from wild carrot progenitors such as Queen Anne’s Lace. These carrots are typically annual, red colored, and characterized by a juicier, coarser texture with larger core sizes. They also require little to no vernalization for flowering (Ellison, 2019; Kulkarni et al., 2023). In contrast, Western carrots (*Daucus carota* ssp. *sativus* var. *sativus*), originated in Northern Europe, are typically biennial, exhibit a deep orange color, and require vernalization for flowering. These carrots have been bred for higher carotene content and possess a more consistent phloem pattern (Ellison, 2019; Kulkarni et al., 2023). Domestication led to significant changes in root morphology, physiology, and biochemical composition, including improved carotenoid content, anthocyanin accumulation, and sugar levels, root size, and reducing lateral root branching. Molecular studies suggest Western varieties likely evolved from Eastern types, highlighting the evolutionary trajectory of carrot domestication (Iorizzo et al., 2013; Baranski et al., 2012; Coe et al., 2023).

In modern plant breeding, molecular markers are crucial in tracking genetic diversity and identifying genes linked to important agronomic traits (Anderson and Lubberstedt, 2003). While random DNA markers, such as AFLPs, detect polymorphisms, recombination events over generations limit their utility. Functional markers (FMs), derived from characterized sequence motifs, offer greater precision in identifying allelic diversity and linking genetic variation to phenotypic expression. Recombination does not affect these markers, making them more reliable for long-term genetic studies (Salgotra and Stewart, 2020). Among these Expressed Sequence Tag-Simple Sequence Repeat (EST-SSR) and Insertion-Deletion (InDel), are PCR-based and co-dominant. InDels, which arise from nucleotide insertions or deletions, play a major role in molecular and chromosomal evolution; second in frequency only after SNPs (Andersson and Purugganan, 2022; Redelings et al., 2024; Savino et al, 2022), providing higher polymorphism than SSRs, enabling detection of subtle genetic variation (Pan et al., 2021). Functionally characterized InDel markers, particularly in or near key genes provide information on key traits such as root size, flowering time, disease resistance, and stress tolerance, aiding marker-assisted selection (Thakur and Randhawa, 2018; Zhao et al., 2018; Shi et al., 2023; Sahu et al., 2017; Cui et al., 2021).

In carrots, InDel-mediated mutations played significant role in domestication and nutritional traits (Rolling et al., 2022). For example, ‘Y’ gene affecting carotenoid accumulation involve 216bp, 2bp, and 60bp in various alleles of the DCAR_032551 gene (Iorizzo et al., 2016), carotene hydroxylase is conditioned with both high total carotenoid content and high alpha-carotene content of carrot roots by an 8bp InDel (Arango et al., 2014; Coe et al., 2021). Sugar composition in edible root is also influenced by a 2.5kb InDel, in the invertase isozyme II Rs gene (Yau and Simon, 2003) and numerous InDels from transposable elements have been associated with intron-length polymorphism in carrots (Grzebelus et al., 2006; Stelmach et al, 2017).

Understanding the genetic diversity within and between cultivated carrot populations is essential for breeding to improve agronomic traits. Population structure analysis, which investigates genetic composition and gene flow, is a powerful tool for understanding the evolutionary forces shaping genetic diversity within a species. Previous studies using SSR markers have revealed significant genetic diversity within cultivated carrot germplasm, with moderate differentiation between Eastern and Western carrot gene pools (Clotault et al., 2010; Baranski et al., 2012; Iorizzo et al., 2013; Chaitra et al., 2020). In this study, we analyzed morphological and molecular diversity between the Eastern and Western carrot gene pools using transcriptome-derived, functionally characterized InDel markers from root and floral tissues (Cholin et al., 2024). This represents the first global report on transcriptome-based InDel marker development and validation in carrots, providing insights into genetic differences among the gene pools and enhancing the use of functional markers in breeding programs.

## Materials and methods

### Plant material

The study included 98 accessions, with 47 accessions representing the Western gene pool from the United States Department of Agriculture (USDA), USA, and 46 representing the Eastern gene pool of Indian origin. Additionally, five check varieties *viz.*, three Eastern types Pusa Rudhira (C1), Pusa Asita (C2), Krishna Prabha Vriddhi (C3), and two Western carrot types Super Kuroda Improved (C4), and Kuroda (C5) were included for agro-morphometric analysis (**Supplementary Table S1**).

### Location of the experiment

Agro-morphometric traits were assessed at two experimental stations in Karnataka, India: (a) the College of Horticulture, Bagalkot (CoH Bagalkot) and (b) the Kittur Rani Channamma College of Horticulture, Arabhavi (KRCCH, Arabhavi). CoH Bagalkot is situated in the northern dry zone at coordinates 16°12′N, 75°45′E, with an average elevation of approximately 610 m and annual rainfall of 552 mm. KRCCH Arabhavi, located in the northern transition zone, is positioned at 16°15′N, 75°45′E, with an elevation of 612.05 m and experiences an average annual precipitation of 554 mm. Both locations have black soil with a loamy texture, ideal for crop growth. The experiment was conducted during the Rabi season (winter cropping season- September to December 2023) using an augmented block design to evaluate 98 accessions, including five check varieties, distributed across 12 blocks.

### Agro-morphometric trait evaluation

Phenotypic observations were recorded on 23 traits, including 10 qualitative (IPGRI,1998 descriptor) and 13 quantitative (**Table 1**), from five randomly selected plants. Biometrical analysis for quantitative traits was performed with the mean data of five plants in each accession.

**Table 1:** Comparison of descriptive statistics across Western and Eastern accession and corresponding check varieties across locations.

Traits	Gene pools/Checks	KRCCH Arabhavi				COH Bagalkot			
		Mean ± SEm	Min.	Max.	CV (%)	Mean ± SEm	Min.	Max.	CV (%)
Days to germination	Western	11.05 ± 0.37	6.0	15.0	20.56	10.95 ± 0.33	6.0	15.0	19.92
	Eastern	6.92 ± 0.27	5.0	12.0	27.86	6.71 ± 0.29	5.0	12.0	28.7
	Check (Western)	8.21 ± 0.21	8.0	8.4	3.62	8.80 ± 0.13	8.7	8.9	2.01
	Check (Eastern)	5.39 ± 0.12	5.2	5.6	3.89	5.89 ± 0.17	5.6	6.2	5.03
Plant Height (cm)	Western	49.77 ± 1.67	30.3	71.0	20.45	41.74 ± 1.55	24.3	63.2	24.27
	Eastern	78.16 ± 1.67	40.3	98.0	15.42	71.7 ± 1.37	44.0	90.0	12.78
	Check (Western)	75.48 ± 0.35	75.1	75.8	0.67	59.35 ± 1.16	58.2	60.5	2.77
	Check (Eastern)	92.16 ± 0.57	91.2	93.1	1.07	74.51 ± 2.31	71.6	79.1	5.38
Number of petioles	Western	9.85 ± 0.5	4.8	17.8	30.84	9.26 ± 0.42	4.9	16.0	29.7
	Eastern	12.68 ± 0.79	7.6	38.0	44.8	9.98 ± 0.35	6.0	15.2	23.19
	Check (Western)	9.70 ± 0.05	9.7	9.8	0.73	9.43 ± 0.20	9.2	9.6	3.05
	Check (Eastern)	11.01 ± 0.46	10.5	11.9	7.2	10.59 ± 0.56	9.6	11.5	9.09
Petiole Length (cm)	Western	32.87 ± 1.44	15.7	51.0	26.67	25.81 ± 1.09	11.5	37.8	27.63
	Eastern	61.05 ± 1.66	28.3	81.0	19.62	53.31 ± 1.23	29.0	70.4	15.48
	Check (Western)	55.77 ± 0.17	55.6	55.9	0.44	38.28 ± 1.43	36.8	39.7	5.29
	Check (Eastern)	71.60 ± 0.31	71.2	72.2	0.75	54.03 ± 3.12	49.5	60.0	10.01
Shoot weight (g)	Western	19.49 ± 1.46	2.3	38.6	45.61	24.18 ± 4.49	1.0	168.0	121.72
	Eastern	103.49 ± 7.16	15.8	291.0	49.85	100.06 ± 5.5	22.0	202.0	36.86
	Check (Western)	60.13 ± 0.08	60.1	60.2	0.18	42.89 ± 3.81	39.1	46.7	12.57
	Check (Eastern)	124.77 ± 3.46	120.9	131.7	4.8	85.30 ± 9.04	72.1	102.6	18.35
Root Length (cm)	Western	16.90 ± 0.45	12.0	23.0	16.03	15.93 ± 0.63	9.7	25.4	26.12
	Eastern	16.93 ± 0.38	12.6	26.0	16.19	18.39 ± 0.45	13.0	26.0	16.58
	Check (Western)	19.71 ± 0.18	19.5	19.9	1.29	21.05 ± 0.27	20.8	21.3	1.81
	Check (Eastern)	20.56 ± 0.30	20.0	20.9	2.52	20.49 ± 0.89	19.1	22.2	7.57
Shoulder length (cm)	Western	2.19 ± 0.12	1.2	5.3	32.5	2.03 ± 0.08	1.2	3.0	24.72
	Eastern	2.32 ± 0.05	1.3	3.3	15.43	3.12 ± 0.1	2.0	5.5	21.35
	Check (Western)	1.00 ± 0.03	1.0	1.0	4.24	1.03 ± 0.01	1.0	1.0	1.6
	Check (Eastern)	2.74 ± 0.05	2.7	2.8	2.99	2.53 ± 0.08	2.4	2.6	5.23
Shoulder Width (cm)	Western	2.31 ± 0.15	1.3	6.3	39.42	1.5 ± 0.07	0.7	2.5	32.35
	Eastern	3.54 ± 0.1	1.6	4.9	21.09	3.7 ± 0.12	2.4	6.3	21.01
	Check (Western)	3.64 ± 0.02	3.6	3.7	0.78	2.76 ± 0.02	2.7	2.8	1.27
	Check (Eastern)	4.06 ± 0.04	4.0	4.1	1.62	3.03 ± 0.29	2.7	3.6	16.59
Root diameter (mm)	Western	23.26 ± 1.24	10.1	48.2	32.42	20.55 ± 1.08	6.4	33.3	34.55
	Eastern	43.84 ± 1.53	26.4	79.9	25.1	29.75 ± 0.92	15.9	39.5	19.85
	Check (Western)	31.80 ± 0.85	31.0	32.7	3.78	35.30 ± 0.40	34.9	35.7	1.61
	Check (Eastern)	38.36 ± 1.41	35.9	40.8	6.35	24.19 ± 2.00	21.1	27.9	14.35
Xylem width (mm)	Western	14.58 ± 1.03	5.3	35.2	42.81	10.99 ± 0.68	3.0	18.6	40.49
	Eastern	35.26 ± 1.41	20.1	72.5	28.78	17.05 ± 0.56	9.2	24.2	21.86
	Check (Western)	20.72 ± 0.74	20.0	21.5	5.05	22.12 ± 1.14	21.0	23.3	7.31
	Check (Eastern)	24.50 ± 0.62	23.4	25.6	4.39	13.62 ± 1.02	12.0	15.5	12.96
Phloem width (mm)	Western	6.18 ± 0.27	2.2	10.5	26.11	4.97 ± 0.25	1.7	8.1	32.57
	Eastern	6.08 ± 0.25	2.7	10.1	29.06	5.79 ± 0.2	3.4	8.9	23.49
	Check (Western)	8.58 ± 0.11	8.5	8.7	1.81	6.98 ± 0.62	6.4	7.6	12.58
	Check (Eastern)	11.37 ± 0.79	10.0	12.7	11.97	4.21 ± 0.42	3.4	4.8	17.32
Root weight (g)	Western	44.12 ± 3.71	12.0	109.8	51.2	36.12 ± 3.58	1.9	74.0	65.07
	Eastern	62.40 ± 3.86	11.6	129.3	44.65	75.21 ± 4.41	26.2	164.0	39.36
	Check (Western)	87.76 ± 1.43	86.3	89.2	2.3	90.71 ± 3.31	87.4	94.0	5.16
	Check (Eastern)	94.97 ± 1.28	92.4	96.4	2.33	48.12 ± 5.48	38.6	57.6	19.71
Days to Maturity	Western	109.70 ± 0.92	104.0	120.0	5.1	110.04 ± 0.77	101.0	120.0	4.56
	Eastern	82.88 ± 0.45	75.0	88.0	3.89	81.62 ± 0.29	76.0	86.0	2.39
	Check (Western)	104.50 ± 0.50	104.0	105.0	0.67	108.95 ± 0.04	108.9	109.0	0.05
	Check (Eastern)	86.66 ± 0.66	86.0	88.0	1.33	82.66 ± 1.20	81.0	85.0	2.51

### Development of InDel markers

For the identification of InDels, twelve RNAseq libraries generated in a previous study (Cholin et al., 2024) for Eastern and Western representative accessions were utilized. Clean reads were obtained by trimming low-quality reads from the raw paired-end FASTQ files, and the reads were then aligned to the DH1 v3.0 carrot reference RNA assembly (Coe et al., 2023; NCBI RefSeq assembly GCF_001625215.2_rna) using the BWA-MEM tool (Li, 2013). BAM files from all 12 libraries were used to generate a BCF file using bcftools mpileup (Li et al., 2009). SNP calling was performed using bcftools call (Li et al., 2009), which generated SNP and InDel information for all 12 samples at each position of the reference RNA. The resulting VCF file, containing InDel information, was used for further analysis. Initially based on the length difference between reference and alternate alleles, 19,817 InDels having >6bp were extracted and functionally annotated (**Supplementary Table S2**). Further, InDel size variations less than 15 bp and heterozygotes were excluded. InDel lengths of 15 bp or longer were considered potential candidates and selected for further evaluation via agarose gel electrophoresis (**Supplementary Table S3**). Flanking sequences for each InDel (0–200 bp upstream and downstream) were extracted from the DH1 v3.0 reference RNA, depending on the position of the InDel. PCR primers were designed using the NCBI Primer-BLAST tool (https://www.ncbi.nlm.nih.gov/tools/primer-blast/) and the Primer3Plus web tool (https://www.primer3plus.com/). The following criteria *viz*., primer length of 18–25 bp, GC content of 40-60%, melting temperature difference between forward and reverse primers of not more than 3°C, and an expected PCR product size of 100–500 bp (Shivaprasad et al., 2024) was considered for designing primer pairs. InDels across 57 important genes of carrots spread across 9 chromosomes were selected for designing primers discussed in the results section (**Supplementary Table S4**). The detailed functions of 57 genes across different organisms along with the references are presented in **Supplementary Table S4** (Aber et al., 2019; Astegno et al., 2017; Baurle et al., 2007; Casson et al., 2009; Chatukuta et al., 2018; Cheng et al., 2022; Dieterle et al., 2005; Eda et al., 2016; Farrow and Facchini, 2014; Ferreyra et al., 2010; Goralogia et al., 2017; Grant et al., 2000; Gruber et al., 2021; Guo et al., 2021; Han et al., 2021, Han et al., 2022; Harshavardhan et al., 2014; Hayama et al., 2019; Inoue et al., 2022; Israel et al., 2022; Janssens and Goris, 2001; Jun et al., 2015; Kotera et al., 2023; Kumar Meena et al., 2019; Li et al., 2021; Liu et al., 2023; Lopez-Ortiz et al., 2020; Marchetti et al., 2020; Masri and Kiss, 2023; Megha et al., 2022; Mishra et al., 2017; Mitsuda et al., 2005; Mousavi et al., 2021; Murray et al., 2006; Nedelyaeva et al., 2020; Nongpiur et al., 2012; North et al., 2007; Oelmuller et al., 1996; Paniagua et al., 2017; Plant et al., 2021; Rempola et al., 2006; Saiga et al., 2008; Saini and Nandi, 2022; Schulz et al., 2013; Seemann et al., 1990; Song et al., 2005; Thanh Ha Thi Do et al., 2018; Thomas et al., 2009; Tzafrir et al., 2002; Valmonte et al., 2014; Waite and Dardick, 2018; Wang et al., 2024; Wang et al., 2005; Wei et al., 2021; Wudick et al., 2018; Yip Delormel and Boudsocq, 2019; Yusa et al., 2019; Zhang et al., 2019; Zhao M. et al., 2022; Zhao Z, 2022).

### Validation of InDel markers

DNA was extracted from the young leaves of a single plant from each of 98 carrot accessions using a modified CTAB extraction protocol (Doyle, 1991). Polymerase chain reaction (PCR) was performed in a reaction mixture containing 50 ng DNA, 2x PCR master mix (AMPLIQON), 0.25 µl each of forward and reverse primer (10 pM), and nuclease-free water. PCR was performed in a thermocycler (Eppendorf India Pvt. Ltd.) with the following conditions: an initial denaturation at 94°C for 5 minutes, followed by 35 cycles of 94°C for 30 seconds, 57–61°C (based on primer GC content) for 45 seconds, and 72°C for 45 seconds, with a final extension at 72°C for 5 minutes. A total of 57 InDel markers designed in the present study were used for genotyping 98 accessions. The amplified fragments were subjected to electrophoresis fractionation in 3.5% agarose gel and visualized by ethidium bromide (EtBr) staining. Clear bands within the expected size range were used for genotypic data generation, with allele sizes (in base pairs) determined using a reference ladder (GeneDirex^®^ 100 bp DNA Ladder RTU).

### Statistical analysis

The mean data of five plants from each accession were used to record agro-morphometric observations at two locations (CoH Bagalkot and KRCCH, Arabhavi) in Karnataka, India. Biometrical analysis was performed for 13 quantitative traits, and the trend was compared across Eastern and Western gene pools. The descriptive statistics analysis and heritability were estimated in the R tool package augmented RCBD (v. 4.4.1). Pearson’s correlation analysis, principal component analysis (PCA), and Euclidean genetic distance-based phylogenetic analysis were performed using PAST 4.03 (Hammer et al., 2001). Twelve qualitative traits were subjected to frequency distribution across Western and Eastern accessions.

The allele scores of polymorphic InDel markers across 98 accessions were subjected to various analyses. Marker diversity parameters and allelic frequencies (Na, Ne, Ho, He, I, F, and PIC), analysis of molecular variance (AMOVA), and principal coordinate analysis (PCoA) were computed using GenAlEx 6.51b2 (Peakall and Smouse, 2012). Each gene pool was treated as a separate population, population-wise marker diversity parameters were compared, and unique or private alleles were extracted from each gene pool. Phylogenetic relationships among the Western and Eastern carrot gene pools were assessed by constructing a neighbor-joining tree using the DARwin 6.0.021 software package (Perrier and Jacquemoud-Collet, 2006) based on Dice’s dissimilarity coefficient. Population structure and gene flow among accessions were examined using a model-based Bayesian clustering approach implemented in STRUCTURE v.2.3.4 (Pritchard et al., 2000). The analysis involved a burn-in period of 10,000 iterations, followed by 10,000 Markov Chain Monte Carlo (MCMC) repetitions. The optimal number of populations (K) was determined by testing K values ranging from 1 to 10, with five independent runs for each K. The true number of populations was identified using the Δ K method (Evanno et al., 2005) by submitting the structure results files to the STRUCTURE SELECTOR website (Earl, 2012), which returned a Delta K value of two. The optimum K value of two was used to assign accessions to populations based on Q values.

Marker-trait association analysis was conducted using phenotypic data from diverse accessions, polymorphic InDel markers and population structure data (Q matrix). The analysis was performed in TASSEL 3.0 (Bradbury et al., 2007) using a general linear model (GLM), with Q as a covariate to account for population structure. Associations were considered highly significant at a threshold of *P* < 0.001.

### Functional characterization of polymorphic InDels

The Gene Ontology (GO) classification analysis was performed in BLAST2GO v6.03 tools using 48 polymorphic markers. The position of InDels was searched for individual genes in NCBI (https://www.ncbi.nlm.nih.gov accessed on 18^th^ November 2024). Further, InDels in the coding region were compared for their amino acid changes in reference and alternate alleles by pairwise alignment in T-COFFEE sequence alignment server (https://tcoffee.crg.eu/apps/tcoffee/do:regular accessed on 29.11.2024).

## Results

### Agro-morphometric traits comparison of Eastern and Western gene pools

**Figure 1:**
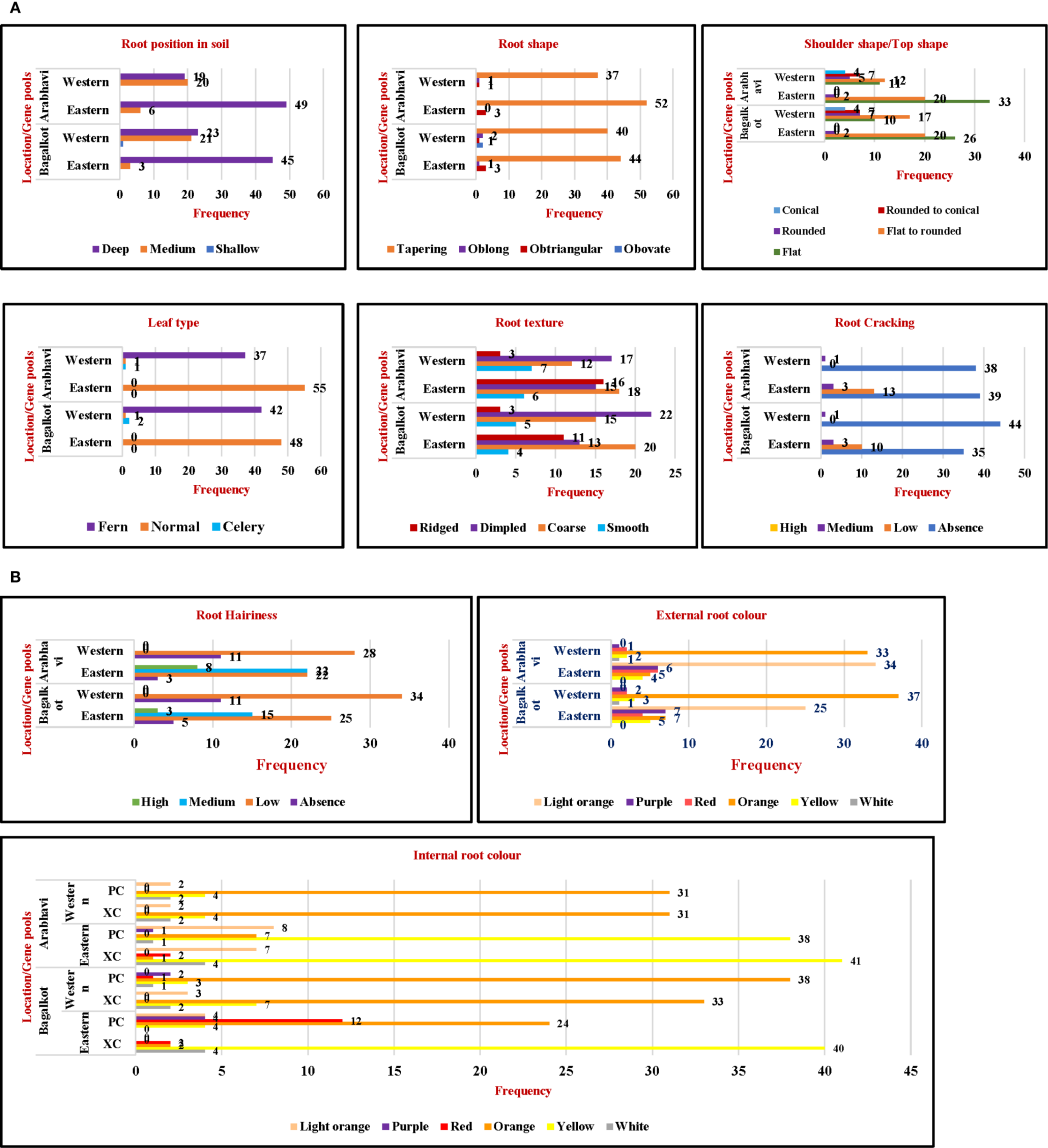
**(A)** Comparative frequency distribution of qualitative traits for two gene pools across locations. **(B)** Comparative frequency distribution of qualitative traits for two gene pools across locations. PC, Phloem color; XC, xylem color.

Qualitative traits such as color, shape, and texture are important parameters when selecting superior accessions for consumer acceptance and breeding programs. Among the 10 qualitative parameters, the expression was consistent across both locations (**Figures 1A, B**). The frequency of medium root position was highest in Western types, while deep root position was most common in Eastern types (**Figure 1A**). For root shape, the majority of the accessions exhibited tapering root shapes (**Figure 1A**). For shoulder shape, Western accessions displayed a predominance of flat to rounded shapes, whereas, Eastern accessions mostly had flat shapes (**Figure 1A**). Western accessions had fern-type leaves, while Eastern types exhibited a normal leaf type (**Figure 1A**). Root texture, hairiness, and cracking are important parameters in carrots influenced by soil conditions and genetic architecture and decide the quality of carrots for a higher price in the market. Among Western accessions, dimpled root texture was predominant, followed by coarse root texture, while in Eastern types, coarse-textured roots were the most common (**Figure 1A**). Cracking was absent in Western accessions, and a few Eastern accessions showed the presence of cracking (**Figure 1A**). Low to medium hairiness was observed in Eastern accessions while Western accessions had low hairiness (**Figure 1B**). Attractive root color is an important parameter in determining consumer acceptance in the market, and carrot carotenoids contribute to consumer health. It is an important parameter that has undergone many genetic changes during primary and secondary domestication events. Western accessions represent the outcome of crop improvement following the domestication event. Consequently, uniformity and consistent expression of both internal and external root colors were key characteristics of Western accessions (**Figure 1B**). The majority of the Western accessions were of orange color with uniform internal and external color, followed by white, yellow, red, and purple hues. In contrast, light orange was the predominant external root color in Eastern accessions, with yellow being the most common internal xylem and phloem color. Few white, purple, and red roots were also found in Eastern accessions, with the lighter intensity of the respective colors in the xylem and phloem showing their distinct vascular tissue pattern (**Figure 1B**).

Descriptive statistics for quantitative traits revealed considerable variation among the 13 quantitative traits across the gene pools, with checks exhibiting less variability (**Table 1**). Among the above-ground traits, the Eastern gene pool demonstrated significantly higher values for plant height (PH), NP, TL, and SW at both locations. Root traits, including root length (RL), top length (TL), TW, RD, XW, and root weight (RW), were also significantly higher in the Eastern gene pool (**Table 1**). Notably, PW, a desirable trait, was higher in Western types at the Arabhavi location. Western carrot types require more time for both days to germination (DG) and days to maturation (DM). Heritability (h²) estimates were higher for most traits, except for root width, root length, phloem width, and root weight in Arabhavi, where these traits exhibited moderate heritability. While in Bagalkot location, higher heritability was observed for the majority of the traits, except for phloem width, number of petioles, and root length, which showed moderate heritability (**Supplementary Table S5**).

**Figure 2:**
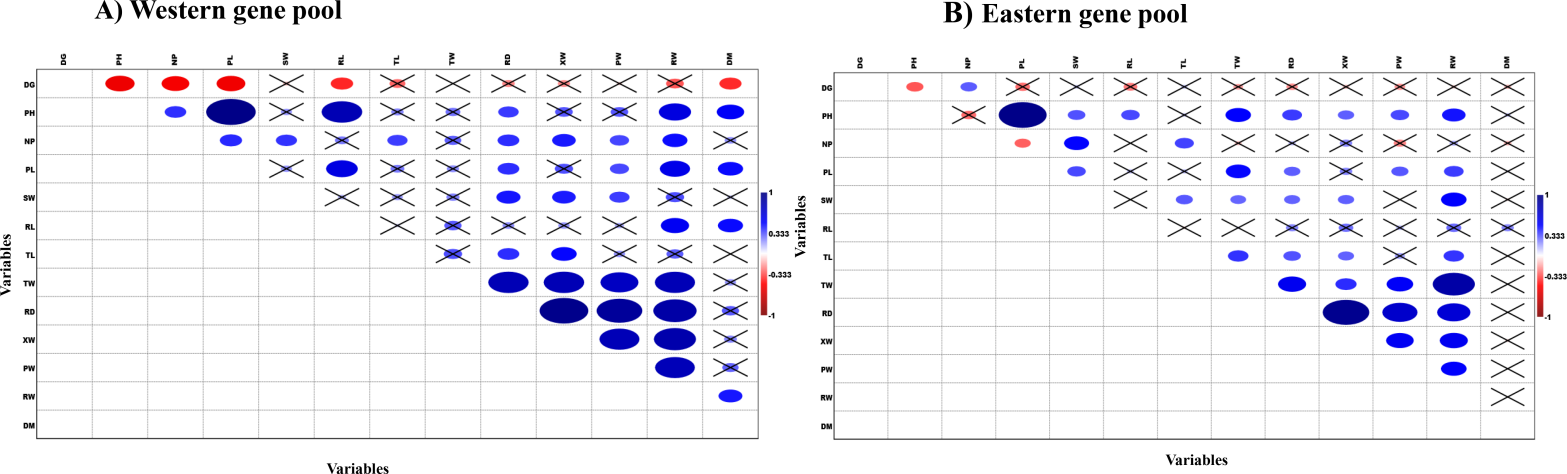
Comparison of correlation pattern among the phenotypic traits for Western and Eastern carrot gene pools. **(A)** Western gene pool. **(B)** Eastern gene pool (DG, Days to germination; PH, Plant height; NP, Number of petioles; PL, Petiole length; SW, Shoot weight; RL, Root length; TL, Top length; TW, Top width; RD, Root diameter; XW, Xylem width; PW, Phloem width; RW, Root weight; DM, Days to maturity).

Pearson’s correlation analysis was performed to examine the relationships between quantitative traits across the Western and Eastern gene pools. The results revealed distinct association patterns between traits in the two gene pools (**Figures 2A, B**). In the Western gene pool, plant height (PH) exhibited a strong positive correlation with petiole length (PL), followed by root length (RL), and a moderate positive correlation with root weight (RW) and days to maturity (DM) (**Figure 2A**). In contrast, in the Eastern gene pool, PH showed a strong positive correlation with PL and was significantly positively correlated with most traits, except for the NP and TL, and DM (**Figure 2B**). SW was positively correlated with RD and XW in the Western gene pool, while in the Eastern gene pool, SW was positively correlated with RW. Additionally, a strong positive correlation was observed between TW, XW, and RD in the Western gene pool. In contrast, TW was positively correlated with RW in the Eastern gene pool. Notably, in the Western gene pool, TW was significantly positively correlated with RD, XW, PW, and RW, whereas in the Eastern gene pool, TW was positively associated with RW and RD. In the Eastern gene pool, RD showed a stronger positive correlation with XW, followed by PW and RW, while in the Western gene pool, XW and PW were similarly positively correlated with RD and RW. For both gene pools, XW showed significant positive correlations with PW and RW, and PW exhibited a positive correlation with RW. These correlation patterns highlight both the shared and distinct relationships between traits across the two gene pools.

**Figure 3:**
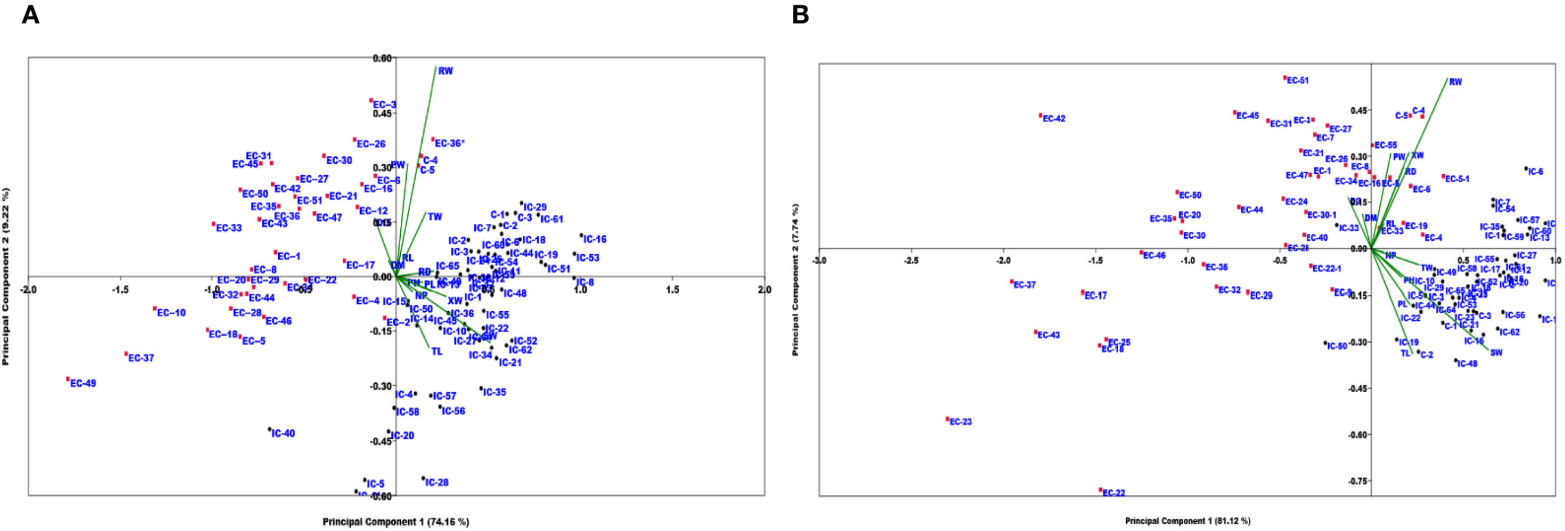
Genotype by trait biplot analysis based on the first two principal components across **(A)** KRCCH Arabhavi location and **(B)** COH Bagalkot location.

Principal component analysis (PCA) of the 13 quantitative traits yielded 13 principal components (**Supplementary Table S6**). The first two components explained more than 80% of the total variance, as shown in the scree plot (**Supplementary Table S6** & **Supplementary Figure S1**). Specifically, the first two components accounted for 74.16% and 9.22% of the variance in Arabhavi, and 81.12% and 7.74% of the variance in Bagalkot, respectively. The first principal component (PC1) explained with majority of variation as observed by the loading scores of 0.986 for shoot weight, 0.825 for plant height, 0.807 for petiole length, 0.787 for xylem width, 0.782 for root diameter, and 0.745 for top length at the Arabhavi location. While, in Bagalkot, PC1 accounted for the majority of variation by shoot weight (0.968), petiole length (0.882), top width (0.881), plant height (0.866), root weight (0.817), and top length (0.803) with their respective loading scores (**Supplementary Tables S6B, C**). The genotype-by-trait (GT) biplot, based on the first two principal components, effectively separated the Eastern and Western accessions at both locations (**Figures 3A, B**). The traits were distributed on the biplot according to their loadings and correlations. In Arabhavi, traits such as PW, RW, RL, TW, RD, DG, and DM formed small vectors with acute angles, indicating strong positive correlations among these traits (**Figure 3A**). In Bagalkot, XW showed positive correlations with these traits, and other vectors with acute angles also reflected positive correlations (**Figure 3B**). Vectors forming angles greater than 90° (e.g., PW and XW, TW and TW in Arabhavi, and SW and PW in Bagalkot) indicated negative correlations. The clear separation of Eastern and Western accessions in distinct quadrants of the biplot highlights their genetic differentiation, with the Western gene pool exhibiting greater genetic diversity than the Eastern gene pool.

**Figure 4:**
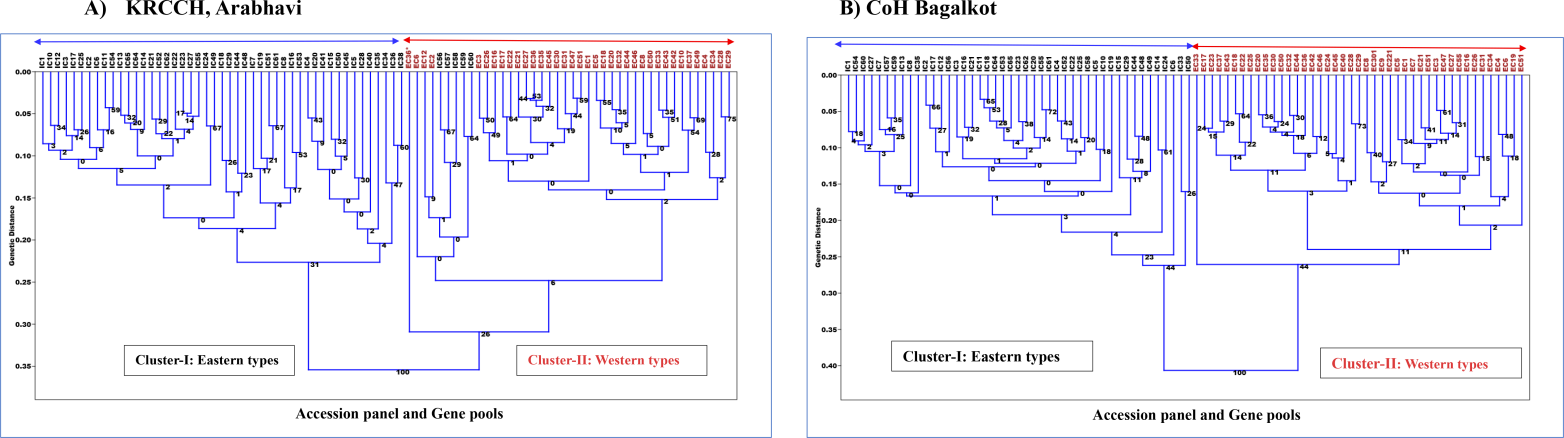
Phylogenetic relationship among Western and Eastern accessions based on quantitative traits in **(A)** KRCCH Arabhavi location and **(B)** COH Bagalkot location (Method: Neighbor-joining tree).

Cluster analysis based on Euclidean distance revealed a clear separation between the Western and Eastern gene pools at both locations (**Figures 4A, B**). At both sites, the phylogenetic tree grouped the accessions into two primary populations, Eastern (cluster1) and Western (cluster2), indicating that substantial variation exists for the two gene pools before and after secondary domestication for these quantitative traits. However, few Eastern accessions (IC-56, IC-57, IC-58, IC-59, IC-60) were observed to be admixtures in the Western group at Arabhavi. This indicates potential gene flow or overlapping traits between the gene pools at this location. The subgroups within the Eastern and Western clusters are due to the genetic variation for these plant growth and root traits among the accessions of each population. This pattern suggests substantial diversity within the individual gene pool.

### Development of InDel markers, primer design, and validation

The RNAseq data were analyzed using reference-based alignment to identify genetic variants, for insertions and deletions (InDels). From 12 paired-end (PE) RNA seq libraries obtained from Eastern and Western representative accessions, a total of 79,468 InDels were identified (data not shown) and initial filtering with the InDel length of >6bp yielded 19,817 InDels and they were functionally annotated with DH1V.3 reference annotation (**Supplementary Table S2**). Further, InDels were filtered to remove all heterozygous variants based on heterozygosity. We then applied a minimum length filter of 15 base pairs (bp), resulting in a refined dataset of 271 homozygous InDels, each 15 bp or longer. **Supplementary Table S3** presents these 271 InDels along with their functional annotations. From these, 57 InDels were chosen for primer design and validation based on their chromosome distribution and association. The developed InDel markers and their corresponding chromosome location, primer sequences, and melting temperature (Tm) are detailed in **Supplementary Table S3**. Detailed information on the position of designed InDels and their function are depicted in **Table 2**.

**Table 2:** Details of list of functionally characterized InDels used for genotyping the diverse set of gene pools with annotated functions, positions and locations in a gene.

Transcript Id	Indel nomenclature	Chr. no.	Gene function	Position	Location
XM_017362999.2	DcFInDel1	7	protein TOPLESS [Daucus carota subsp. sativus]	3936	3’UTR
XM_017380084.2	DcFInDel2	2	disease resistance protein RPM1 [Daucus carota subsp. sativus]	3032	3’UTR
XM_017367168.2	DcFInDel3	8	flowering time control protein FCA isoform X1 [Daucus carota subsp. sativus]	3460	CODING
XM_017359659.2	DcFInDel5	7	cyclic dof factor 1 [Daucus carota subsp. sativus]	107	5’UTR
XM_064087627.1	DcFInDel6	1	photosystem II CP43 reaction center protein [Daucus carota subsp. sativus]	19188	3’UTR
XM_017381146.2	DcFInDel7	2	cullin-3A [Daucus carota subsp. sativus]	2757	CODING
XM_017402326.2	DcFInDel9	6	SART-1 family protein DOT2 [Daucus carota subsp. sativus]	324	CODING
XM_017363833.2	DcFInDel10	7	CBL-interacting serine/threonine-protein kinase 25 [Daucus carota subsp. sativus]	1581	3’UTR
XM_017395960.2	DcFInDel11	5	protein IQ-DOMAIN 8 [Daucus carota subsp. sativus]	1298	3’UTR
XM_017375770.2	DcFInDel12	2	F-box/kelch-repeat protein At1g26930 [Daucus carota subsp. sativus]	230	5’UTR
XM_064079953.1	DcFInDel13	6	zinc finger CCCH domain-containing protein 53 [Daucus carota subsp. sativus]	3126	3’UTR
XM_017361287.2	DcFInDel14	7	protein BIG GRAIN 1-like E [Daucus carota subsp. sativus]	1234	CODING
XM_017380433.2	DcFInDel16	2	ABC transporter B family member 20 [Daucus carota subsp. sativus]	4583	3’UTR
XR_001806615.2	DcFInDel17	5	protein LITTLE ZIPPER 3 [Daucus carota subsp. sativus]	423	3’UTR
XM_017375321.2	DcFInDel18	1	biotin carboxylase 1, chloroplastic [Daucus carota subsp. sativus]	1967	3’UTR
XM_017400085.2	DcFInDel19	6	NAC domain-containing protein 41 [Daucus carota subsp. sativus]	729	CODING
XM_017363643.2	DcFInDel20	1	BURP domain protein RD22 [Daucus carota subsp. sativus]	268	CODING
XM_064090015.1	DcFInDel21	1	ubiquitin carboxyl-terminal hydrolase 18 [Daucus carota subsp. sativus]	3223	CODING
XM_017362733.2	DcFInDel22	1	protein TILLER ANGLE CONTROL 1 [Daucus carota subsp. sativus]	707	CODING
XM_017376294.2	DcFInDel24	2	protein cornichon homolog 4-like [Daucus carota subsp. sativus]	724	3’UTR
XM_017381112.2	DcFInDel25	2	putative clathrin assembly protein At2g01600 isoform X1 [Daucus carota subsp. sativus]	2113	3’UTR
XM_064088842.1	DcFInDel26	3	cytochrome P450 734A1-like isoform X1 [Daucus carota subsp. sativus]	1235	CODING
XM_017382015.2	DcFInDel27	3	glutathione gamma-glutamylcysteinyltransferase 1 [Daucus carota subsp. sativus]	163	5’UTR
XM_017385820.2	DcFInDel28	3	calcium-dependent protein kinase SK5 [Daucus carota subsp. sativus]	1801	3’UTR
XM_017386390.2	DcFInDel29	3	probable 2-carboxy-D-arabinitol-1-phosphatase isoform X1 [Daucus carota subsp. sativus]	1747	3’UTR
XM_017388126.2	DcFInDel31	4	probable aquaporin PIP2–4 [Daucus carota subsp. sativus]	1032	3’UTR
XM_017388311.2	DcFInDel32	4	carboxyl-terminal-processing peptidase 3, chloroplastic [Daucus carota subsp. sativus]	1665	3’UTR
XM_017392478.2	DcFInDel33	4	transmembrane emp24 domain-containing protein p24delta3 [Daucus carota subsp. sativus]	76	3’ UTR
XM_017392040.2	DcFInDel34	4	calcium-dependent protein kinase 1 [Daucus carota subsp. sativus]	445	5’ UTR
XM_017388045.2	DcFInDel35	4	protein ABA DEFICIENT 4, chloroplastic-like [Daucus carota subsp. sativus]	1311	3’ UTR
XM_017394094.2	DcFInDel37	5	probable calcium-binding protein CML36 [Daucus carota subsp. sativus]	850	5’UTR
XM_017396789.1	DcFInDel38	5	serine/threonine protein phosphatase 2A 59 kDa regulatory subunit B’ gamma isoform [Daucus carota subsp. sativus]	2418	5’UTR
XR_001807669.2	DcFInDel40	6	histidine kinase 1-like isoform X1 [Daucus carota subsp. sativus]	756	3’UTR
XM_017401473.2	DcFInDel41	6	rho GDP-dissociation inhibitor 1 [Daucus carota subsp. sativus]	942	3’UTR
XM_017360262.2	DcFInDel42	7	acyl carrier protein 1, mitochondrial [Daucus carota subsp. sativus]	593	3’UTR
XM_017363925.2	DcFInDel43	7	zinc finger protein BRUTUS-like At1g74770 [Daucus carota subsp. sativus]	3826	CODING
XM_017360436.2	DcFInDel44	7	dirigent protein 22 [Daucus carota subsp. sativus]	656	CODING
XM_017362876.2	DcFInDel45	7	protein ESSENTIAL FOR POTEXVIRUS ACCUMULATION 1 isoform X2 [Daucus carota subsp. sativus]	4016	CODING
XM_017359816.2	DcFInDel46	7	BTB/POZ domain-containing protein At1g67900 [Daucus carota subsp. sativus]	2500	CODING
XM_017367089.2	DcFInDel48	8	2-oxoglutarate-Fe(II) type oxidoreductase hxnY [Daucus carota subsp. sativus]	1112	3’UTR
XM_017367382.2	DcFInDel49	8	paired amphipathic helix protein Sin3-like 4 [Daucus carota subsp. sativus]	3822	3’UTR
XM_017365115.2	DcFInDel50	8	pentatricopeptide repeat-containing protein At5g41170, mitochondrial-like isoform X1 [Daucus carota subsp. sativus]	2297	5’UTR
XM_017368157.2	DcFInDel51	8	ankyrin repeat-containing protein At2g01680-like [Daucus carota subsp. sativus]	1598	CODING
XM_017367962.2	DcFInDel52	8	large ribosomal subunit protein uL10 [Daucus carota subsp. sativus]	1295	3’UTR
XM_017368719.2	DcFInDel53	9	pentatricopeptide repeat-containing protein At5g09450, mitochondrial [Daucus carota subsp. sativus]	1488	3’UTR
XM_017371676.2	DcFInDel54	9	benzyl alcohol O-benzoyltransferase [Daucus carota subsp. sativus]	257	CODING
XM_017369462.2	DcFInDel55	9	F-box protein At1g55000 [Daucus carota subsp. sativus]	989	3’UTR
XM_017371413.2	DcFInDel56	9	protein OBERON 3 [Daucus carota subsp. sativus]	156	5’ UTR

These markers were further validated and utilized for diversity analysis across 98 accessions, representing both the Eastern and Western gene pools. Among these, 48 InDels were found to be polymorphic (84.21%), and they were used for assessing diversity and population structure across the gene pools. The physical locations of the 271 functional InDels identified in this study are depicted along with the mapped genes, QTLs associated with domestication traits, and selective sweeps across nine haploid chromosomes of carrot (**Supplementary Figure S1**). These QTLs helped to select the genes associated with the domestication traits and further aid in *de novo* domestication studies.

### Gene ontology of polymorphic InDels

GO classification of the 48 polymorphic InDels provided insights into their potential biological functions (**Supplementary Table S6**). A total of 72 biological processes (BP), 25 cellular components (CC), and 40 molecular function (MF) terms were assigned to 26, 24, and 24 genes, respectively. In BP terms, ‘Cellular’ and ‘metabolic’ processes were most common. While in CC ‘cellular anatomical structure’ and ‘intercellular anatomical structure’ were predominant and in MF terms ‘binding’ and ‘activity’ terms were most common. This functional annotation suggests that these InDels may be involved in a diverse range of biological processes, including metabolic processes, cellular component organization, and catalytic activity. These findings highlight the potential of these InDels as valuable markers for improving carrot breeding programs. For instance, InDels in Y locus genes regulating carotenoid biosynthesis alter enzyme expression and metabolite accumulation, affecting root color, antioxidant capacity, and nutrition (Iorizzo et al., 2016). Indels in cellulose or pectin biosynthetic genes modify cell wall properties, impacting cell expansion and root morphology. Changes in enzyme or motif-binding sites due to InDels disrupt carotenoid accumulation and further influence root structure (Liu et al., 2019).

### Molecular diversity parameters

**Figure 5:**
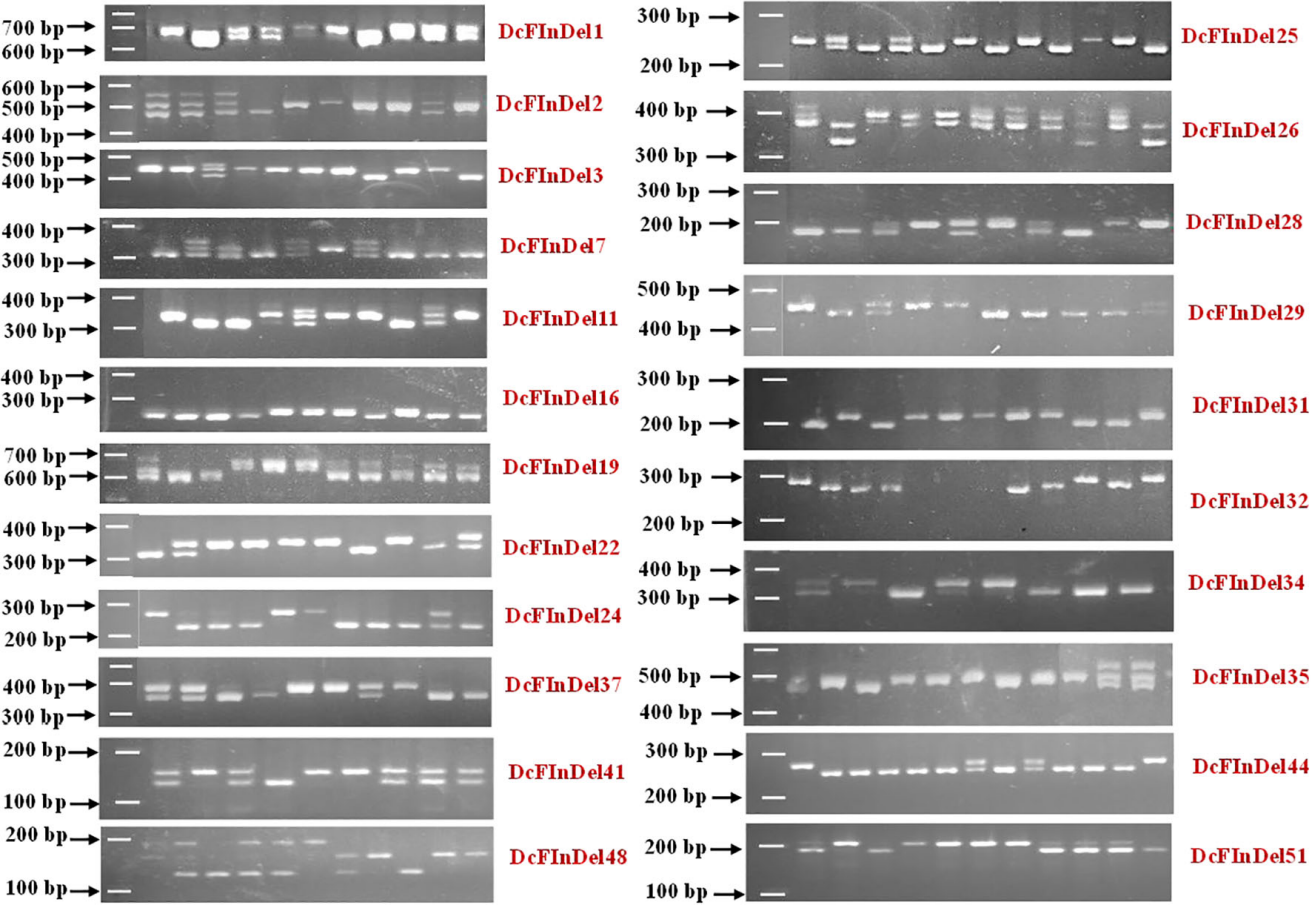
Representative profiles of InDel markers developed and validated across gene pools. Control images are shown here for illustrative purposes with original gels shown in **Figure 3A**.

Out of 57 validated InDels, 48 markers demonstrated polymorphism across 98 accessions, yielding a polymorphic percentage of 84.21%. This indicates that the in-house transcript data utilized to develop these InDels is highly effective for investigating the Eastern and Western gene pools. The remaining 9 InDels were found to be monomorphic across the panel but different from the DH reference genome. The representative polymorphic InDel variations across gene pools are presented in **Figure 5**.

A total of 224 alleles were identified from 98 accessions across the 48 markers, with the Western gene pool contributing a higher number of alleles (113) in comparison to the Eastern population (111). This difference highlights changes that may have occurred after domestication, likely resulting in improved traits within the Western population (**Table 3**). The Western population harbored a higher number of private alleles (13 alleles) compared to the Eastern population (11 alleles) (**Table 4**). These unique alleles reflect genetic modifications that may have influenced the function of corresponding genes both before and after domestication within these ancestrally related gene pools.

**Table 3:** Comparison of allelic diversity of 48 polymorphic InDels across Western and Eastern gene pools.

Sl. no.	Locus	Na		Ne		I		Ho		He		F		PIC
		Western	Eastern	Western	Eastern	Western	Eastern	Western	Eastern	Western	Eastern	Western	Eastern	
1	DcFInDel1	2.00	2.00	1.53	1.87	0.53	0.66	0.19	0.30	0.35	0.47	0.45	0.35	0.369
2	DcFInDel2	2.00	2.00	1.78	1.04	0.63	0.10	0.22	0.00	0.44	0.04	0.51	1.00	0.243
3	DcFInDel3	2.00	2.00	1.27	1.74	0.37	0.62	0.12	0.36	0.21	0.43	0.43	0.15	0.279
4	DcFInDel5	2.00	2.00	1.68	1.90	0.59	0.67	0.50	0.35	0.40	0.47	-0.24	0.25	0.346
5	DcFInDel6	4.00	4.00	3.60	2.90	1.33	1.18	1.00	1.00	0.72	0.65	-0.38	-0.53	0.698
6	DcFInDel7	2.00	3.00	1.38	1.56	0.45	0.58	0.29	0.10	0.28	0.36	-0.05	0.71	0.272
7	DcFInDel9	2.00	2.00	1.94	1.18	0.68	0.29	0.00	0.00	0.49	0.15	1.00	1.00	0.311
8	DcFInDel10	2.00	4.00	1.04	1.87	0.10	0.86	0.00	0.40	0.04	0.47	1.00	0.15	0.266
9	DcFInDel11	2.00	2.00	2.00	1.83	0.69	0.65	0.11	0.04	0.50	0.45	0.79	0.91	0.369
10	DcFInDel12	3.00	1.00	1.88	1.00	0.73	0.00	0.65	0.00	0.47	0.00	-0.39	#NA	0.258
11	DcFInDel13	2.00	2.00	1.33	1.06	0.42	0.14	0.17	0.02	0.25	0.06	0.33	0.66	0.147
12	DcFInDel14	3.00	3.00	2.88	1.90	1.08	0.78	0.31	0.40	0.65	0.47	0.53	0.16	0.535
13	DcFInDel16	2.00	2.00	1.51	1.35	0.52	0.43	0.10	0.18	0.34	0.26	0.70	0.29	0.255
14	DcFInDel17	2.00	2.00	1.04	1.06	0.10	0.14	0.04	0.06	0.04	0.06	-0.02	-0.03	0.050
15	DcFInDel18	2.00	1.00	1.10	1.00	0.19	0.00	0.10	0.00	0.09	0.00	-0.05	#NA	0.043
16	DcFInDel19	2.00	2.00	1.73	1.82	0.61	0.64	0.30	0.34	0.42	0.45	0.28	0.24	0.342
17	DcFInDel20	2.00	2.00	1.29	1.07	0.38	0.14	0.13	0.06	0.22	0.06	0.43	-0.03	0.136
18	DcFInDel21	2.00	2.00	1.17	1.41	0.27	0.47	0.02	0.23	0.14	0.29	0.85	0.21	0.374
19	DcFInDel22	2.00	2.00	1.88	1.65	0.66	0.58	0.20	0.23	0.47	0.39	0.56	0.41	0.373
20	DcFInDel24	1.00	2.00	1.00	1.76	0.00	0.62	0.00	0.28	0.00	0.43	#NA	0.35	0.345
21	DcFInDel25	2.00	2.00	1.72	1.53	0.61	0.53	0.21	0.16	0.42	0.35	0.49	0.53	0.373
22	DcFInDel26	3.00	4.00	2.70	2.41	1.05	1.01	0.91	0.40	0.63	0.58	-0.45	0.31	0.574
23	DcFInDel27	2.00	1.00	1.52	1.00	0.53	0.00	0.15	0.00	0.34	0.00	0.57	#NA	0.164
24	DcFInDel28	4.00	5.00	2.96	1.47	1.18	0.70	0.22	0.15	0.66	0.32	0.66	0.53	0.597
25	DcFInDel29	3.00	3.00	2.20	1.90	0.89	0.74	0.16	0.22	0.55	0.47	0.70	0.54	0.449
26	DcFInDel31	2.00	3.00	1.63	1.29	0.57	0.46	0.15	0.17	0.39	0.23	0.62	0.26	0.398
27	DcFInDel32	3.00	3.00	2.60	1.97	1.02	0.82	0.11	0.06	0.62	0.49	0.82	0.89	0.506
28	DcFInDel33	1.00	2.00	1.00	1.38	0.00	0.45	0.00	0.24	0.00	0.27	#NA	0.10	0.139
29	DcFInDel34	2.00	2.00	1.21	1.98	0.32	0.69	0.07	0.31	0.18	0.49	0.63	0.37	0.342
30	DcFInDel35	3.00	2.00	1.48	1.59	0.56	0.56	0.23	0.36	0.32	0.37	0.29	0.02	0.295
31	DcFInDel37	2.00	2.00	1.14	1.88	0.24	0.66	0.13	0.25	0.12	0.47	-0.07	0.47	0.367
32	DcFInDel38	4.00	3.00	2.25	1.41	0.99	0.56	0.08	0.00	0.56	0.29	0.85	1.00	0.401
33	DcFInDel40	5.00	2.00	2.38	1.61	1.14	0.57	0.28	0.27	0.58	0.38	0.52	0.30	0.486
34	DcFInDel41	2.00	2.00	1.74	1.32	0.62	0.40	0.32	0.09	0.43	0.24	0.25	0.61	0.370
35	DcFInDel42	1.00	2.00	1.00	1.40	0.00	0.46	0.00	0.14	0.00	0.29	#NA	0.50	0.147
36	DcFInDel43	1.00	2.00	1.00	1.11	0.00	0.21	0.00	0.11	0.00	0.10	#NA	-0.06	0.044
37	DcFInDel44	2.00	2.00	1.81	1.51	0.64	0.52	0.31	0.14	0.45	0.34	0.31	0.58	0.320
38	DcFInDel45	2.00	2.00	1.76	1.30	0.62	0.39	0.33	0.22	0.43	0.23	0.23	0.06	0.352
39	DcFInDel46	2.00	2.00	1.94	1.52	0.68	0.53	0.23	0.23	0.49	0.34	0.52	0.33	0.365
40	DcFInDel48	3.00	3.00	2.83	2.87	1.07	1.07	0.40	0.41	0.65	0.65	0.37	0.37	0.589
41	DcFInDel49	4.00	2.00	2.81	1.11	1.20	0.20	0.22	0.10	0.64	0.10	0.66	-0.05	0.385
42	DcFInDel50	2.00	2.00	1.79	2.00	0.63	0.69	0.34	0.33	0.44	0.50	0.23	0.35	0.369
43	DcFInDel51	2.00	2.00	1.98	1.88	0.69	0.66	0.61	0.62	0.50	0.47	-0.23	-0.32	0.373
44	DcFInDel52	4.00	3.00	1.57	2.01	0.70	0.80	0.10	0.26	0.36	0.50	0.71	0.49	0.386
45	DcFInDel53	2.00	2.00	1.15	1.76	0.25	0.62	0.14	0.00	0.13	0.43	-0.07	1.00	0.304
46	DcFInDel54	3.00	3.00	2.00	1.37	0.87	0.53	0.10	0.12	0.50	0.27	0.81	0.55	0.485
47	DcFInDel55	2.00	2.00	1.43	1.06	0.48	0.14	0.19	0.06	0.30	0.06	0.39	-0.03	0.170
48	DcFInDel56	2.00	2.00	1.65	1.69	0.58	0.60	0.29	0.32	0.39	0.41	0.26	0.22	0.321
	Mean ± SEm	2.35 ± 0.13	2.31 ± 0.11	1.76 ± 0.09	1.59 ± 0.06	0.59 ± 0.05	0.52 ± 0.04	0.22 ± 0.03	0.21 ± 0.03	0.37 ± 0.03	0.33 ± 0.03	0.38 ± 0.06	0.36 ± 0.05	0.335

Na, Number of alleles; Ne, Number of effective alleles; I, Shannon’s information index; Ho, Observed heterozygosity; He, Expected heterozygosity; F, Fixation index; PIC, Polymorphic information content; SEm, Standard error of means.

**Table 4:** Private/unique alleles and their frequency across Western and Eastern genepools from 48 functional InDels.

Gene pool	Locus	Allele	Frequency	Gene pool	Locus	Allele	Frequency
Western	DcFInDel12	250	0.326	Eastern	DcFInDel7	358	0.010
	DcFInDel12	260	0.023		DcFInDel10	388	0.083
	DcFInDel18	820	0.048		DcFInDel10	420	0.198
	DcFInDel27	92	0.220		DcFInDel24	237	0.685
	DcFInDel35	550	0.021		DcFInDel26	395	0.143
	DcFInDel38	416	0.042		DcFInDel28	200	0.074
	DcFInDel40	160	0.064		DcFInDel31	228	0.042
	DcFInDel40	168	0.053		DcFInDel33	102	0.163
	DcFInDel40	194	0.053		DcFInDel42	270	0.173
	DcFInDel49	297	0.195		DcFInDel43	285	0.054
	DcFInDel49	308	0.098		DcFInDel53	251	0.317
	DcFInDel52	414	0.042				
	DcFInDel53	310	0.069				

The Western population also exhibited a higher average number of effective alleles (1.76 ± 0.09) compared to the Eastern population (1.59 ± 0.06). Similarly, the Western population showed a higher average Shannon-Weaver index (‘I’) of 0.59 ± 0.05, compared to 0.52 ± 0.04 in the Eastern population, suggesting greater overall genetic diversity. Observed heterozygosity (Ho) was also higher in the Western gene pool (0.22 ± 0.03) than in the Eastern gene pool (0.21 ± 0.03), further indicating greater genetic variation within the Western population. Expected heterozygosity (He) was higher in the Western population (0.37 ± 0.03) compared to the Eastern population (0.33 ± 0.03), reinforcing the trend of greater diversity in the Western gene pool. The fixation index (F) ranged from -0.53 (DcFInDel24), indicating an excess of heterozygotes, to 1.00 (DcFInDel33), revealing complete fixation. In contrast, loci DcFInDel12 and DcFInDel27 showed no variation in the Eastern population, suggesting domestication-driven diversification in the Western types.

The polymorphic information content (PIC) values for the polymorphic markers ranged from 0.043 to 0.698, with an average PIC of 0.335. The highest PIC value was observed in the marker DcFInDel6, indicating its higher informativeness. Markers with a PIC greater than 0.50, such as DcFInDel6 and DcFInDel28, were highly informative and polymorphic, making them particularly effective for distinguishing individual accessions. The distinct allelic diversity was evident in both the gene pools for 16 InDel markers (DCFInDel7, 10, 12, 18, 20, 26, 27, 28, 33, 35, 38, 40, 42, 43, 49, and 52), suggesting their utility in the study of domestication. These results highlight the complex genetic landscape of carrots and provide valuable insights into the genetic differentiation and domestication of the species.

### Population structure, AMOVA and molecular phylogenetic analysis

**Figure 6:**
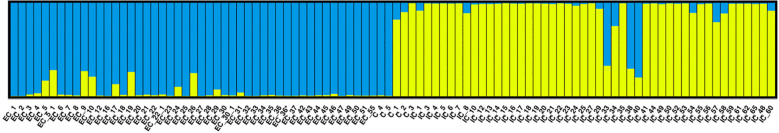
Population structure analysis showing the classification of 98 accessions of Western and Eastern gene pools and checks considering K = 2 allowing admixture.

Population structure analysis was performed on the 98 accessions using the STRUCTURE 2.3.3 tool to gain insights into the genetic relationships among gene pools. The optimal K value of two, based on ΔK, indicated a clear division of accessions into two primary populations (**Supplementary Figure S3**). Cluster-1 represented the Western gene pool, while cluster-2 represented the Eastern gene pool (**Figure 6**). Within the Western population, few accessions (EC-5, EC-5-1, EC-9, EC-10, EC-17, EC-19, EC-24, and EC-26) were found as admixtures with Eastern types, suggesting that these accessions may have the potential to adapt to tropical conditions and exhibit minimal genetic dissimilarity to the Eastern gene pool. Conversely, within the Eastern population, accessions IC-33, IC-34, IC-38, and IC-40 were major admixtures with the Western types, indicating that these accessions likely originated as selections from the Western pool as confirmed by their phenotypic observations similar to Western cultivars.

**Figure 7:**
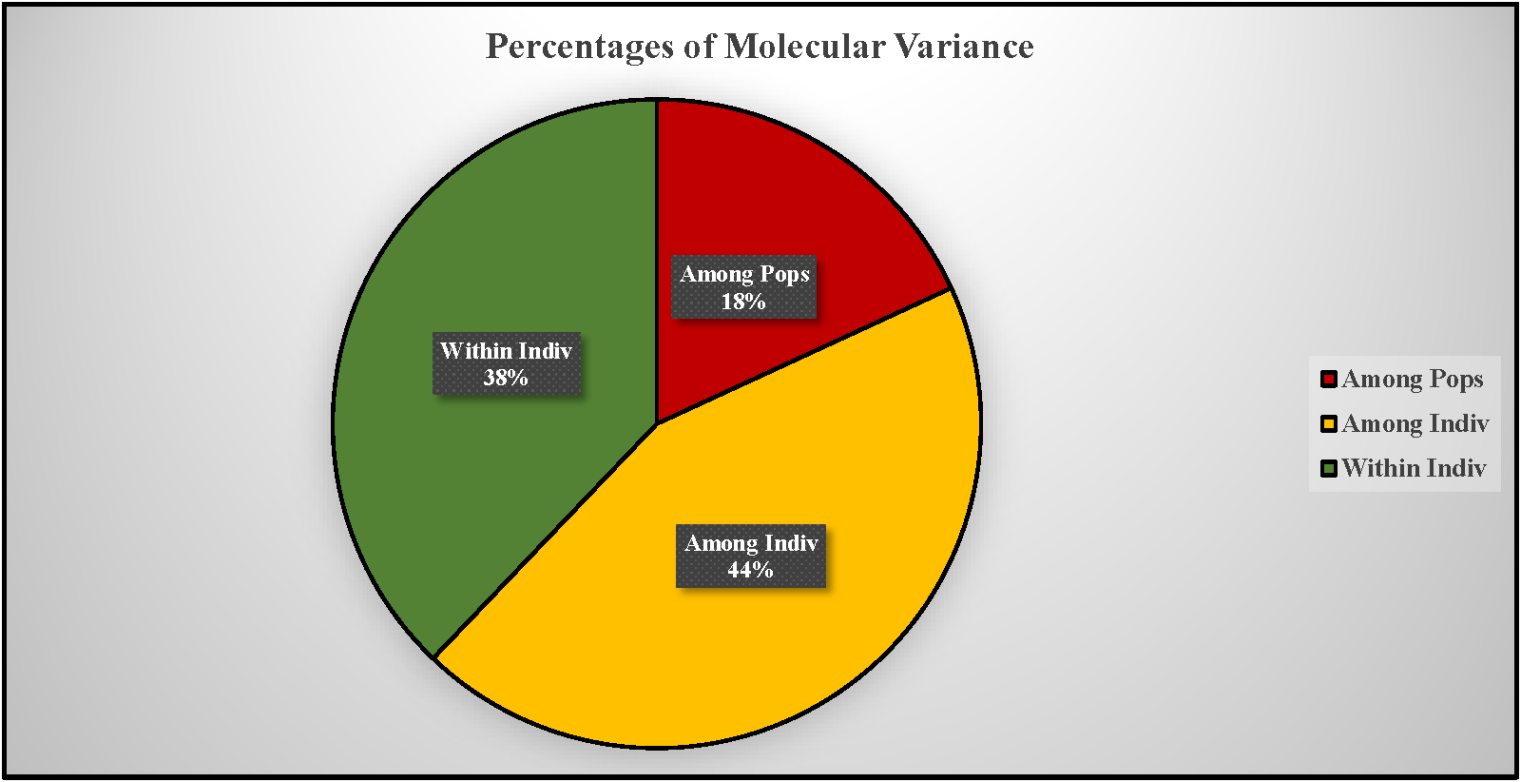
Analysis of molecular variance (AMOVA) showing the contribution of various components to total molecular variance from two populations/gene pools.

**Figure 8:**
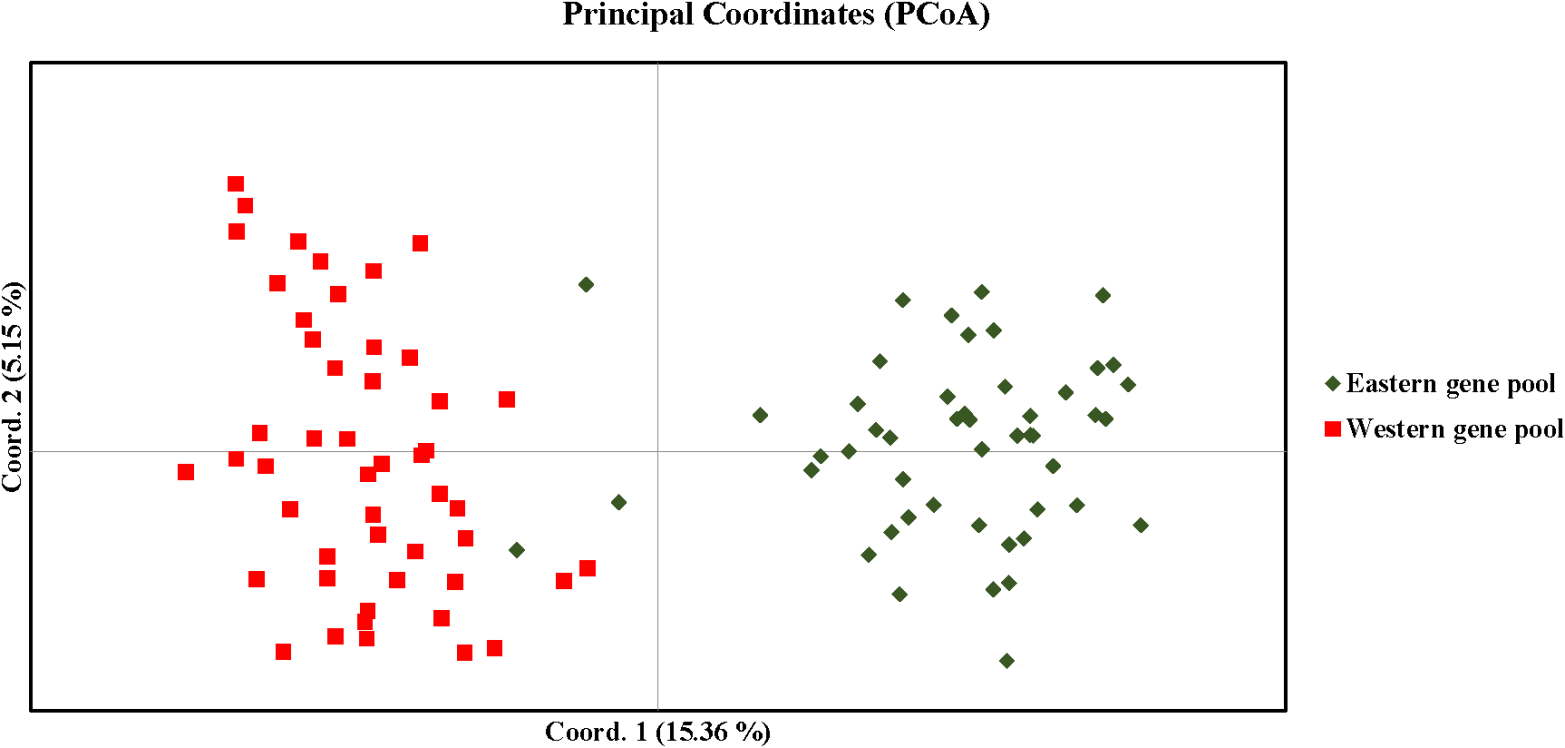
Distribution of accessions in different co-ordinates of PCoA (Principal co-ordinate analysis).

AMOVA revealed significant genetic differentiation among the accessions, with 18% of the total variance attributed to differences between populations. The majority of the variance (44%) was observed among individuals within the two populations, while 38% of the variance occurred within individuals across the entire population (**Figure 7**). F-statistics further indicated substantial genetic diversity within the population, with a fixation index relative to the total population (Fit) of 0.621. The fixation indices for population differentiation (Fst) and within-population genetic structure (Fis) were 0.181 and 0.537, respectively. The pairwise Fst value of 0.127 and pairwise Nm value of 1.716 respectively suggest a moderate genetic differentiation and higher gene flow between the populations as expected for the highly outcrossing nature of carrots (**Table 5**). Principal coordinate analysis (PCoA) effectively separated the accessions into distinct Western and Eastern groups, with the first two principal coordinates explaining 15.36% and 5.15% of the variance, respectively (**Figure 8**). Eastern accessions IC-33, IC-38, and IC-40 were identified as admixtures within the Western population, suggesting that these accessions likely originated from selections within the Western gene pool.

**Table 5:** Pairwise *Fst* (above diagonal) and Nm (below diagonal).

Gene pools	Western	Eastern
Western	0.000	0.127
Eastern	1.716	0.000

**Figure 9:**
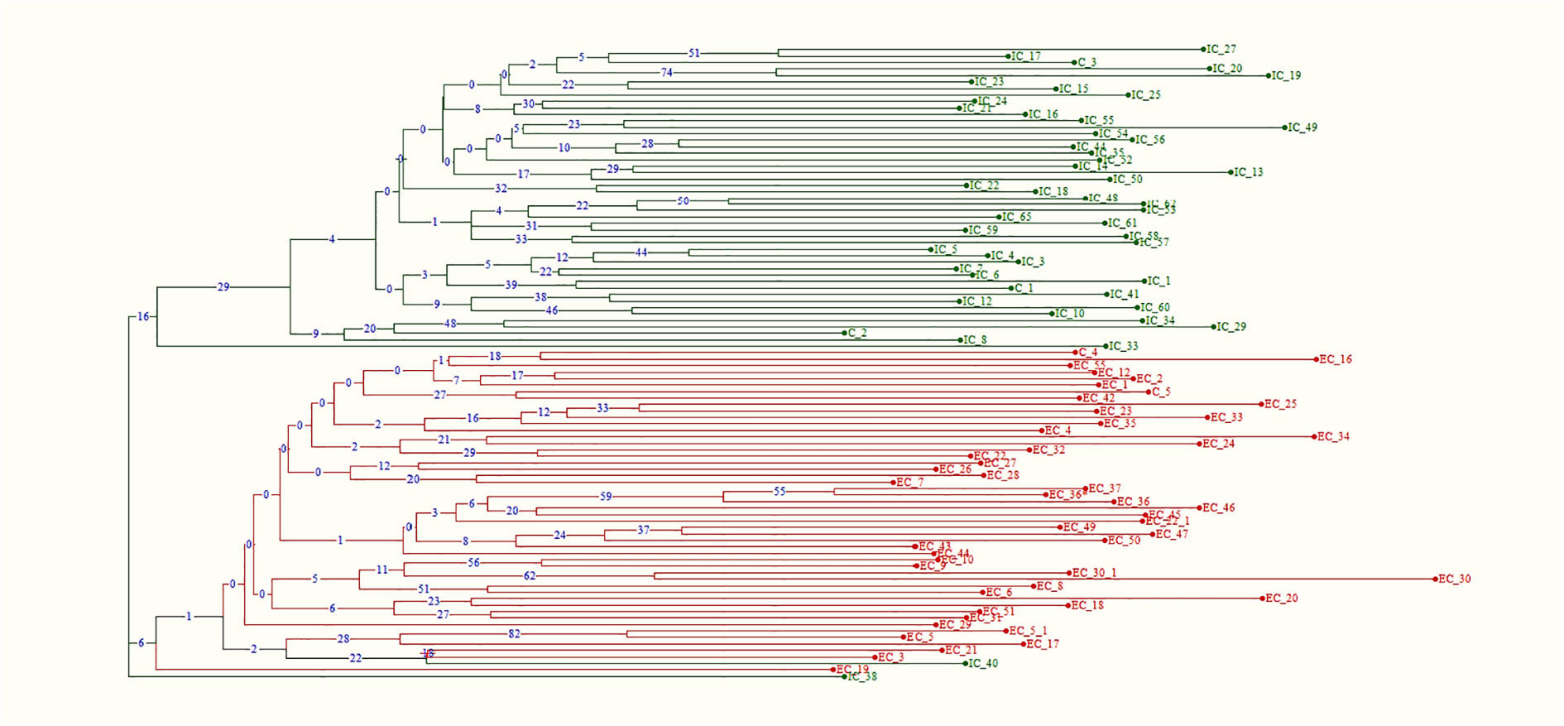
Neighbor-joining tree depicting phylogenetic relationship among Western and Eastern gene pool using 48 polymorphic functional markers (boot strap-1000).

Phylogenetic analysis, based on Dice’s genetic dissimilarity matrix, revealed that the 98 accessions were grouped into three distinct clusters (**Figure 9**). The first cluster consisted of Eastern accessions and Eastern checks, while the second cluster included Western accessions and Western checks. Notably, the Eastern accession IC-40 was classified as an admixture within the Western population, suggesting that it shares phenotypic similarities with Western types and may have originated from selections within the Western gene pool. The third cluster comprised the Eastern genotype IC-38, which formed a solitary group, indicating that it is genetically distinct from both the Eastern and Western populations. This analysis highlights the complex genetic relationships and potential gene flow between the two gene pools, as well as the presence of genetically unique accessions.

### Marker-trait association

At the Arabhavi location, significant marker-trait associations were identified for five quantitative and five qualitative parameters. Markers DcFInDel55, DcFInDel28, and DcFInDel38 exhibited substantial contributions to RW (R² = 0.21), SW (R² = 0.18), and SL (R² = 0.10), respectively, with high phenotypic variance explained (PVE). For qualitative traits, marker DcFInDel32 showed strong associations with RP (R² = 0.21) and RS (R² = 0.30), while DcFInDel28 also significantly influenced RS with a higher R² of 0.32. In addition, markers DcFInDel10, DcFInDel55, and DcFInDel38 contributed to RC (R² = 0.26), LT (R² = 0.13), and SS (R² = 0.21), respectively, highlighting their role in these traits.

At CoH Bagalkot, a significant association was found for three quantitative and five qualitative traits. DcFInDel7 and DcFInDel55 demonstrated notable contributions to PH (R² = 0.05) and XW (R² = 0.13), respectively. Moreover, markers DcFInDel26 and DcFInDel28 together accounted for 0.18 PVE in TW. Among qualitative traits, DcFInDel32 was associated with both RP (R² = 0.34) and RS (R² = 0.35) with high PVE, while DcFInDel10, DcFInDel28, and DcFInDel49 were significantly associated with RC, RS, and SS, with R² values of 0.24, 0.29, and 0.17, respectively (**Table 6**). Several markers had consistent associations across both locations for the same traits. Notably, DcFInDel10 was associated with RC, DcFInDel32 with both RP and RS, and DcFInDel28 with RS. These markers, particularly DcFInDel32, which was associated with both RP and RS at both locations, are promising candidates for further validation and application in marker-assisted selection (MAS) to improve carrot breeding programs.

**Table 6:** Significant marker-trait associations for root traits (both quantitative and qualitative traits) across both the locations using general linear model.

Quantitative traits				
Location	Trait	Marker	Marker p	markerR^2^
KRCCH Arabhavi	Days to germination	DcFIndel3	8.16×10^-4^	0.08
	Days to maturity	DcFIndel26	8.75×10^-4^	0.07
	Root diameter (mm)	DcFIndel55	8.69×10^-5^	0.21
	Shoulder length	DcFIndel38	1.05×10^-4^	0.10
	Shoot weight	DcFIndel28	1.34×10^-5^	0.18
CoH Bagalkot	Plant height (cm)	DcFIndel7	4.11×10^-4^	0.05
	Shoulder width (mm)	DcFIndel26	1.06×10-^5^	0.10
		DcFIndel28	6.22×10^-4^	0.08
	Xylem width (mm)	DcFIndel55	3.33×10^-4^	0.13
Qualitative traits				
Location	Trait	Marker	Marker p	markerR^2^
KRCCH Arabhavi	Root Cracking	DcFIndel10	7.86×10^-6^	0.26
	Leaf type	DcFIndel55	1.23×10^-4^	0.13
	Root position	DcFIndel32	8.78×10^-4^	0.21
	Root shape	DcFIndel28	1.37×10-^4^	0.32
		DcFIndel32	7.62×10^-4^	0.30
	Shoulder shape	DcFIndel38	2.98×10-^4^	0.21
CoH Bagalkot	Root Cracking	DcFIndel10	2.40×10-^5^	0.24
	Leaf type	DcFIndel20	1.05×10^-4^	0.07
	Root position	DcFIndel32	2.61×10^-7^	0.34
	Root shape	DcFIndel28	4.85×10-^4^	0.29
		DcFIndel32	1.44×10^-4^	0.35
	Shoulder shape	DcFIndel49	9.79×10^-4^	0.17

### Functional characterization of validated InDels

Among 48 polymorphic InDels assessed for their position in the gene, 15 InDels were found in the coding region, eight were in 5’UTR, and 25 were in 3’UTR (**Table 2**). The amino acid changes due to insertions or deletions in the coding sequences of 15 genes were assessed (**Supplementary Figure S4**). The changes in the nucleotide sequences at InDel regions of coding sequences significantly changed the amino acid composition, predicting the change in gene function and/or expression level across the gene pools. Further validation through gene expression analysis confirms their functionality and role in evolutionary changes during secondary domestication.

## Discussion

Eastern carrots originated in Central Asia, while Western carrots were introduced to Europe and underwent further improvements during the post-domestication era (Ellison, 2019). During domestication, specific alleles were differentially selected in Eastern and Western carrots. Hence, Western and Eastern accessions vary in many domesticated traits, including plant growth habits, root development characteristics, flowering behavior, carotenoid accumulation, vernalization requirement, root quality (Ellison, 2019). Previous studies elucidated domestication changes and allelic diversity among diverse accessions (Iorizzo et al., 2013; Rong et al., 2014; Coe et al., 2023). However, the specific genetic mechanisms driving domestication changes and the genotypic variability causing phenotypic changes are still unclear. In the present investigation, morphological and molecular variation was evaluated and characterized using agro-morphometric and functional InDel markers to understand the genetic variations of important phenotypic and molecular changes across evolutionarily related Eastern and Western gene pools of two diverse geographic regions of the world.

### Diversity in agro-morphometric patterns

Agro-morphological traits exhibited distinct variations between Western and Eastern carrot gene pools. Western accessions predominantly displayed tapering roots with dimpled textures, while Eastern types exhibited coarser textures and varied root positioning. Leaf type also differed, with Western accessions showcasing a fern leaf type and Eastern accessions exhibiting broad, typical carrot leaves. These phenotypic divergences are likely adaptations to specific regional growing conditions. Temperate climates often have colder winters and shorter growing seasons. We speculate that fern-type leaves may have evolved as a way to reduce the plant’s surface area, more efficiently capture light, minimize heat and enable water loss during colder periods. Root color, a crucial trait influencing market value and consumer preference, varied significantly between the two pools. Western accessions exhibited a wide range of colors, including orange, white, yellow, red, and purple, while Eastern accessions predominantly displayed light orange roots. These color variations are associated with genetic differences in carotenoid and anthocyanin biosynthesis and accumulation pathways.

Vegetative parameters also differed, with Eastern types exhibiting higher plant height, number of petioles, and petiole length, indicative of a stronger source-sink relationship. However, this increased vegetative growth was linked to a larger top length and width, a less desirable trait. In contrast, Western types, more sensitive to day length, showed delayed germination, and growth however, a more compact plant architecture with limited shoulder development. Root characters revealed further distinctions. Eastern types displayed a larger shoulder (top or crown), contributing significantly to root weight. Additionally, this was accompanied by a higher xylem width, negatively impacting consumer preference. Western types, on the other hand, had a negligible shoulder and a higher proportion of phloem tissue, resulting in a more desirable root shape and smooth texture (Luby et al., 2016). Genetic factors influencing vascular tissue development, such as MYB15, WRKY46, and AP2/ERF TF, may be crucial targets for improving root quality in Eastern carrot varieties (Kulkarni et al., 2023). Heritability estimates for most traits were notably high (> 60%) as depicted in **Supplementary Table S5**, indicating a strong genetic influence on their expression suggesting their high amenability to genetic improvement through selective breeding (Luby et al., 2016).

Pearson’s correlation analysis revealed distinct trait associations within the Western and Eastern gene pools. While both pools shared some common relationships like positive correlation between plant height and petiole length, they also exhibited unique patterns. For instance, Western accessions had a stronger link between shoot weight and root diameter, while Eastern accessions exhibited a stronger association between shoot weight and root weight and distinct xylem and phloem contribution towards root weight. Higher top width, length, plant height, and petiole length in Eastern accessions may have contributed to increased xylem width, facilitating a more efficient reproductive switch. In contrast, Western accessions, with their higher photosynthetic efficiency and lower xylem content, exhibited superior root quality and enhanced beta-carotene accumulation (Cholin et al., 2024).

PCA and cluster analysis further underscored the genetic divergence between the two gene pools. The GT-biplot separated Eastern and Western accessions, highlighting their distinct genetic makeup. The cluster analysis corroborated this finding, grouping accessions into distinct clusters based on genetic similarity (Singh et al., 2021). These findings suggest that the Western and Eastern gene pools have undergone distinct evolutionary trajectories, leading to the development of unique trait combinations (Stelmach et al., 2021; Hadagali et al., 2025). The presence of greater phenotypic variation among the Western gene pool makes it amenable to crop improvement (Luby et al., 2016). The identification of these genetic differences among the phenotypic traits provides valuable insights into the genetic architecture of carrots and has significant implications for future breeding programs. By understanding the genetic basis of these traits, breeders can develop cultivars with improved yield, quality, and stress tolerance. For instance, targeting the genetic loci associated with root length and phloem width, root texture, early germination, and maturity could lead to the development of high-yielding, superior-quality carrot cultivars.

### Functional characterization of InDels and their diversity across gene pools

Exploration of the transcriptome data from Eastern and Western carrot accessions has led to the identification of a significant number of InDel variants. The distribution of these InDels across different chromosomes suggests a complex genetic architecture underlying carrot domestication. These InDels, particularly those associated with key genes related to domestication, offer valuable insights into the genetic diversity and evolutionary history of carrots. Important carrot genes, such as root development, flowering, physiological processes, biotic stress, and abiotic stress tolerance genes that played a critical role in trait modification during domestication, were selected. Eastern and western carrot accessions can be easily distinguished by a few important domestication traits/markers.

Using 57 InDel markers, molecular analysis revealed 48 polymorphic markers, with a high percentage of polymorphism (84.21%), suggesting that these markers are highly informative and suitable for assessing genetic diversity across carrot accessions. The validation of a set of 48 polymorphic InDel markers provides powerful and efficient tools for genetic diversity and population structure studies. The Western gene pool exhibited higher genetic diversity, as evidenced by the greater number of alleles, unique alleles, and higher values of genetic diversity indices. Higher allele count in the Western gene pool may be due to the introduction of new alleles after the domestication event, owing to evolutionary forces. This suggests that the Western gene pool has undergone a more intense selection and genetic diversification during the domestication, and crop improvement period (Ellison, 2019; Luby et al., 2016; Coe et al., 2023; Kulkarni et al., 2023). The results of the present study contrast with earlier reports by Iorizzo et al. (2013); Coe et al. (2023) and Chaitra et al. (2020), which found higher genetic divergence in Eastern than in the Western gene pool, highlighting the domestication bottleneck. The difference may be attributed to marker type, as SNPs primarily detect single-nucleotide substitutions and SSRs target tandem repeats, in contrast, the functional InDels employed here are more effective in capturing domestication-associated structural changes. The present study underscores the functional significance of genetic variations, specifically InDels, across ancestrally related Eastern and Western carrot gene pools. By analyzing these variations, we hypothesize that the Western gene pool, having undergone more intensive selection and improvement programs, exhibits greater genetic diversity and phenotypic variation. Even though the accessions of the two diverse gene pools were collected from geographically distinct locations (India and the USA), the moderate Fst and high Nm values suggest a moderate level of genetic differentiation and significant gene flow between the populations. This indicates a shared evolutionary history and potential for genetic exchange between the two gene pools.

Molecular phylogeny, population structure analysis, and PCoA further clarified the genetic relationships between the accessions. The STRUCTURE analysis, revealing two distinct populations corresponding to the Western and Eastern gene pools, aligns with phenotypic observations. This underscores the importance of integrating both morphological and molecular data in assessing genetic diversity. The observed admixture in certain accessions, such as IC-40 and EC-5, suggests potential gene flow between the two gene pools, likely due to historical crossbreeding or the movement of accessions across regions. Previous studies have utilized InDel markers to genetically group accessions in other crops like Cannabis (Pan et al., 2021), and radish (Wang et al., 2015; Li et al., 2023).

The identification of significant marker-trait associations provides valuable insights into the genetic architecture of key carrot traits. Markers such as DcFInDel10, DcFInDel28, DcFInDel32, DcFInDel38, and DcFInDel55 were consistently associated with multiple traits across different environments with significant phenotypic variance, highlighting their potential as robust markers for marker-assisted selection (MAS). For instance, DcFInDel28, linked to root shape and shoot weight, is annotated as calcium-dependent protein kinase SK5 (CDPKSK5), which regulates root growth, development, and stress response. DcFInDel32, associated with carboxyl-terminal-processing peptidase 3 (ctp3), plays a vital role in chloroplast development, photosynthesis, and overall plant development. Together, these markers explain over 50% of the variance (R²) in root shape, indicating their potential for carrot improvement. DcFInDel10, a marker for root cracking, is annotated as CBL-interacting serine/threonine-protein kinase 25 (CIPK25), involved in regulating abiotic stress responses. DcFInDel55, associated with root width and leaf type, is annotated as F-box protein At1g55000, which regulates cell cycle, development, flowering, stress responses, and hormonal signaling. These markers offer valuable tools for improving root shape, reducing cracking, and enhancing yield in carrot breeding.

Identifying InDels within coding and regulatory regions highlights their potential impact on gene expression and protein function in carrots. We further hypothesize that these genetic variations have played a crucial role in the diversification of carrot cultivars. GO classification also revealed their association with key biological functions, including cellular and metabolic processes, growth, development, and responses to stresses. Understanding the functional implications of these genetic variants provides opportunities for breeders to exploit them as functional markers in targeted breeding aimed at enhancing carrot yield, quality, and stress tolerance.

## Conclusion

This is the first study to generate and validate carrot InDels, and apply them to dissect agro-morphometric and molecular diversity between Eastern and Western gene pools. Using transcriptome-derived InDels, we effectively demonstrated significant genotypic differences across two distinct gene pools. A clear genotypic difference was observed, with Eastern varieties exhibiting larger plant size and root dimensions, while Western varieties possessed deeper root position and uniform deep orange color. The InDel markers displayed high polymorphism, revealing strong genetic differentiation between the two gene pools, which underscores their potential for utilizing both gene pools in breeding programs. These functional InDels represent valuable molecular resources for understanding the evolutionary divergence and accelerating breeding through marker-assisted selection, enabling the development of high-yielding, climate-resilient cultivars with superior quality.

## Data Availability

The datasets presented in this study can be found in online repositories. The names of the repository/repositories and accession number(s) can be found in the article/**Supplementary Material**.
