# Supplementary material for: Exploring functional InDels and genetic diversity: agro-morphometric and molecular insights into the Western and Eastern gene pools of carrot (Daucus carota L.)
Source: Front Plant Sci. 2025 Oct 22;16:1658653. doi: 10.3389/fpls.2025.1658653 (PMC12587770; doi:10.3389/fpls.2025.1658653)
Supplement: Supplementary file 1 [file DataSheet1.zip › Supplementary Figure5_PDF Mudihal et al.pdf]

Figure S4: Functional InDel influenced amino acid changes in the coding region of genes

Note: ref: reference sequence; alt-alternate sequence with insertion or deletion
a) DcFInDel3 - XM_017367168.2 (flowering time control protein FCA isoform X1)

```
DcFInDel3_ref_V   MDRHRGGAADRYGDSRPYRNSRAPPPPPPRGDGAYPMKKHHNNHNHTSPDGSYFNSGFSSGGR
DcFInDel3_alt_V   MDRHRGGAADRYGDSRPYRNSRAPPPPPPRGDGAYPMKKHHNNHNHTSPDGSYFNSGFSSGGR
cons              ***************************************************************

DcFInDel3_ref_V   DRGGVNSPPRFSGGGGGGRGPGFRGGGGRGGSGNFDQRSPNGGGGRGGMGNFDKQGPGSGGGR
DcFInDel3_alt_V   DRGGVNSPPRFSGGGGGGRGPGFRGGGGRGGSGNFDQRSPNGGGGRGGMGNFDKQGPGSGGGR
cons              ***************************************************************

DcFInDel3_ref_V   GGAGNFDQRGPSGGGGRGNFDHQGPPASGGGYGPGNFDQQGVVGGGERGNFDYHGPITGGGYG
DcFInDel3_alt_V   GGAGNFDQRGPSGGGGRGNFDHQGPPASGGGYGPGNFDQQGVVGGGERGNFDYHGPITGGGYG
cons              ***************************************************************

DcFInDel3_ref_V   HGNFDQQGPISGGERGNFDHQGPVSRGGRGGVVNYDEQGPVSVGGRGGMSNFDQQGPISGRAH
DcFInDel3_alt_V   HGNFDQQGPISGGERGNFDHQGPVSRGGRGGVVNYDEQGPVSVGGRGGMSNFDQQGPISGRAH
cons              ***************************************************************

DcFInDel3_ref_V   GDFDQQGPLSGGARGNFEQQDSVSGGGRGNFDRQGPISGGRGNFDQQGPVSGVGRGNFDQQGP
DcFInDel3_alt_V   GDFDQQGPLSGGARGNFEQQDSVSGGGRGNFDRQGPISGGRGNFDQQGPVSGVGRGNFDQQGP
cons              ***************************************************************

DcFInDel3_ref_V   VSGGGRGGAVNFDHQGPVISGGRGGAVNFDQQGPTTGGGRGGRVDFDPQGPVTGHKRGYPFSA
DcFInDel3_alt_V   VSGGGRGGAVNFDHQGPVISGGRGGAVNFDQQGPTTGGGRGGRVDFDPQGPVTGHKRGYPFSA
cons              ***************************************************************

DcFInDel3_ref_V   PAVSPDNQDGGAFAKLFVGSVPRTATEEDIRPLFDQHGRVLEVALIKDKKTGQQQGCCFIKYA
DcFInDel3_alt_V   PAVSPDNQDGGAFAKLFVGSVPRTATEEDIRPLFDQHGRVLEVALIKDKKTGQQQGCCFIKYA
cons              ***************************************************************

DcFInDel3_ref_V   TSGEADRAIRALHNQHTLPGGVGPIQVRYADGERERLGAVEYKLFVGSLNKQAIEKEVEEIFS
DcFInDel3_alt_V   TSGEADRAIRALHNQHTLPGGVGPIQVRYADGERERLGAVEYKLFVGSLNKQAIEKEVEEIFS
cons              ***************************************************************

DcFInDel3_ref_V   PYGRVEDVYLMRDEMKQSRGCGFVKYSSRESAMAAINSLSGSYIMRGCDQPLTVRFADPKRPR
DcFInDel3_alt_V   PYGRVEDVYLMRDEMKQSRGCGFVKYSSRESAMAAINSLSGSYIMRGCDQPLTVRFADPKRPR
cons              ***************************************************************

DcFInDel3_ref_V   PGESRNGPSFGGPGFGPRFQPPGLRPMPNTIETTHNHNLPNTWHPMSPQNQVPSDVGIHTRFP
DcFInDel3_alt_V   PGESRNGPSFGGPGFGPRFQPPGLRPMPNTIETTHNHNLPNTWHPMSPQNQVPSDVGIHTRFP
cons              ***************************************************************

DcFInDel3_ref_V   APSTPGGSSSGGFSGSADGSSPVFPVSSSTVPQNYNQSKPQVPSFSQQITPVQQQPYHSSQQY
DcFInDel3_alt_V   APSTPGGSSSGGFSGSADGSSPVFPVSSSTVPQNYNQSKPQVPSFSQQITPVQQQPYHSSQQY
cons              ***************************************************************

DcFInDel3_ref_V   PSSMQSQSAGSYPQKQTSVAQPTQS----------IQLGAQTPYSQTYSSQKQPGVNGQLPVS
DcFInDel3_alt_V   PSSMQSQSAGSYPQKQTSVAQPTQSIQLGAQPTQSIQLGAQTPYSQTYSSQKQPGVNGQLPVS
cons              *************************          **************************************

DcFInDel3_ref_V   LSHNQQNLPPASTQIPSNNNVPPQSLPGIANQPQLNPQQQFPQPLHQSPSQLTAQMLSQQTQA
DcFInDel3_alt_V   LSHNQQNLPPASTQIPSNNNVPPQSLPGIANQPQLNPQQQFPQPLHQSPSQLTAQMLSQQTQA
cons              ***************************************************************

DcFInDel3_ref_V   LQARLQSSQQAFSQIQQQLQMMQPSNQSFTMQQGPKASSQQTSWDGMTPQTSASSKVNPPVAD
DcFInDel3_alt_V   LQARLQSSQQAFSQIQQQLQMMQPSNQSFTMQQGPKASSQQTSWDGMTPQTSASSKVNPPVAD
cons              ***************************************************************

DcFInDel3_ref_V   GPSAATAPSVIPEMTHTAVPLKCNWTEHTSPEGFKYYYNSTTGESKWEKPEELSSFEKQQTQL
DcFInDel3_alt_V   GPSAATAPSVIPEMTHTAVPLKCNWTEHTSPEGFKYYYNSTTGESKWEKPEELSSFEKQQTQL
cons              ***************************************************************

DcFInDel3_ref_V   QKSSVQQPQSQFQPQGLPTQQVPPNPQGQFQPQLQPQLRYPPQLQQPSQSSSYQAPGYAGHQG
DcFInDel3_alt_V   QKSSVQQPQSQFQPQGLPTQQVPPNPQGQFQPQLQPQLRYPPQLQQPSQSSSYQAPGYAGHQG
cons              ***************************************************************
```

```
DcFInDel3_ref_V    TQDIAYKQSPAVASSVNDPSRFQQGLQGSQEW
DcFInDel3_alt_V    TQDIAYKQSPAVASSVNDPSRFQQGLQGSQEW
cons               ********************************
```

b) DcFInDel9 - XM_017402326.2 (SART-1 family protein DOT2)

```
DcFInDel9_ref_V    MDRDDSPARERWEGGHDDLEDGGELTRDSSRHRSTKDSGKTSRRDDKTRHRSKDPERSKELEK
DcFInDel9_alt_V    MDRDDSPARERWEGGHDDLEDGGELTRDSSRHRSTKDSGKT-------RHRSKDPERSKELEK
cons               *****************************************       ***************

DcFInDel9_ref_V    ERVSSRDRRKEGRDEYSKREKPRDKDGDVDKYRERDRERDRDRKDYGKDKERERERDSLKDSE
DcFInDel9_alt_V    ERVSSRDRRKEGRDEYSKREKPRDKDGDVDKYRERDRERDRDRKDYGKDKERERERDSLKDSE
cons               ***************************************************************

DcFInDel9_ref_V    RGHEKDRGKDRSKDRDKEKEKDRDARGKDREREKEREKHKGREDREKEILDREKVKDRVRDKE
DcFInDel9_alt_V    RGHEKDRGKDRSKDRDKEKEKDRDARGKDREREKEREKHKGREDREKEILDREKVKDRVRDKE
cons               ***************************************************************

DcFInDel9_ref_V    REVNMDKERSRERDKVSRKQRDDGHERSKDIVTDDKLNSEADDVHYRDSTKQVTGLHTGADDA
DcFInDel9_alt_V    REVNMDKERSRERDKVSRKQRDDGHERSKDIVTDDKLNSEADDVHYRDSTKQVTGLHTGADDA
cons               ***************************************************************

DcFInDel9_ref_V    RNLKLDKNASTEPVASHTAASEIKERISRMKEERLKKTSEGSSDILSWVNKSRKIEEKRNAEK
DcFInDel9_alt_V    RNLKLDKNASTEPVASHTAASEIKERISRMKEERLKKTSEGSSDILSWVNKSRKIEEKRNAEK
cons               ***************************************************************

DcFInDel9_ref_V    ERALQLSRVFEEQDNINQGDSDDEAAAQHTSHDLSGVKVLHGLDKVIEGGAVVLTLKDQSILA
DcFInDel9_alt_V    ERALQLSRVFEEQDNINQGDSDDEAAAQHTSHDLSGVKVLHGLDKVIEGGAVVLTLKDQSILA
cons               ***************************************************************

DcFInDel9_ref_V    NGDLNEDVDMLENVEIGEQKQRDDAYKAAKKKPGTYEDKFSDDLGTEKKMLPQYDDPVADEGV
DcFInDel9_alt_V    NGDLNEDVDMLENVEIGEQKQRDDAYKAAKKKPGTYEDKFSDDLGTEKKMLPQYDDPVADEGV
cons               ***************************************************************

DcFInDel9_ref_V    TLDAAGRFGGEAKKKLDELRRRIEGVSAASHFEDLDSSAKTSSDYYTHEEMLRFKKPKKKKSL
DcFInDel9_alt_V    TLDAAGRFGGEAKKKLDELRRRIEGVSAASHFEDLDSSAKTSSDYYTHEEMLRFKKPKKKKSL
cons               ***************************************************************

DcFInDel9_ref_V    RKKDKLDLDALEAEAVSSGLGVNDLGSRSNGTRQALKEEQEKSEAEKRSSAFQSAYAKAAEAS
DcFInDel9_alt_V    RKKDKLDLDALEAEAVSSGLGVNDLGSRSNGTRQALKEEQEKSEAEKRSSAFQSAYAKAAEAS
cons               ***************************************************************

DcFInDel9_ref_V    NALRPERRATSQPMEEDDDTVFGDDDDDLRKSLERARKLALEKQAESAPSGPQAIAIIASSTA
DcFInDel9_alt_V    NALRPERRATSQPMEEDDDTVFGDDDDDLRKSLERARKLALEKQAESAPSGPQAIAIIASSTA
cons               ***************************************************************

DcFInDel9_ref_V    TKSGADNENPLSGDPQENKVVFTEMEEFVWGLQLGEEAHKPEGEDVFMEEDEAPEASVGEKKA
DcFInDel9_alt_V    TKSGADNENPLSGDPQENKVVFTEMEEFVWGLQLGEEAHKPEGEDVFMEEDEAPEASVGEKKA
cons               ***************************************************************

DcFInDel9_ref_V    EDGGWTEMQDTAKDEIPSKDKQEDIALDETIHEVAVGKGLSGALNLLKDRGALKESVEWGGRN
DcFInDel9_alt_V    EDGGWTEMQDTAKDEIPSKDKQEDIALDETIHEVAVGKGLSGALNLLKDRGALKESVEWGGRN
cons               ***************************************************************

DcFInDel9_ref_V    MDKKKSKLVGISDNDGKKEIRIERTDEYGRILTPKEAFRLISHKFHGKGPGKMKQEKRMRQYQ
DcFInDel9_alt_V    MDKKKSKLVGISDNDGKKEIRIERTDEYGRILTPKEAFRLISHKFHGKGPGKMKQEKRMRQYQ
cons               ***************************************************************

DcFInDel9_ref_V    EELKVKKMKNSDTPSLSVERMREAQAQLKTPYLVLSGNVKPGQTSDPRSGFATVEKDLPGGLT
DcFInDel9_alt_V    EELKVKKMKNSDTPSLSVERMREAQAQLKTPYLVLSGNVKPGQTSDPRSGFATVEKDLPGGLT
cons               ***************************************************************

DcFInDel9_ref_V    PMLGDRKVEHFLGIKRKSDPGDMGPPKRPKPEG
DcFInDel9_alt_V    PMLGDRKVEHFLGIKRKSDPGDMGPPKRPKPEG
cons               *********************************
```

c) DcFInDel14 - XM_017361287.2 (protein BIG GRAIN 1-like E)

```
DcFInDel14_ref_    MSIQQVPERIWKKSFHNRRDSDELDVFEAAGYFSGANEVSGYQKMLRSGRRTSLDIPMMSSTR
DcFInDel14_alt_    MSIQQVPERIWKKSFHNRRDSDELDVFEAAGYFSGANEVSGYQKMLRSGRRTSLDIPMMSSTR
cons               ***************************************************************

DcFInDel14_ref_    TSMIQEQAHQYDNVIMPEKPTKEKKYKQPNSPGGRLATFLNSLFSQNSSKKKKSKSCSAQSMK
DcFInDel14_alt_    TSMIQEQAHQYDNVIMPEKPTKEKKYKQPNSPGGRLATFLNSLFSQNSSKKKKSKSCSAQSMK
cons               ***************************************************************

DcFInDel14_ref_    DEDHESPGARRKRRSSISHFRSFTSTNNSSSVNNSKSSFYSSSSSEFTTHPSTNTPTKNTYKD
DcFInDel14_alt_    DEDHESPGARRKRRSSISHFRSFTSTNNSSSVNNSKSSFYSSSSSEFTTHPSTNTPTKNTYKD
cons               ***************************************************************

DcFInDel14_ref_    SKPTIHLNMSSKSNKYNVNSTTWQKNEDHDNVWMDEEEFKFSTTTIFDSNRAPSQNREIFGNG
DcFInDel14_alt_    SKPTIHLNMSSKSNKYNVNSTTWQKNEDHDNVWMDEEEFKFSTTTIFDSNRAPSGNGAF----
cons               ****************************************************** *  :

DcFInDel14_ref_    AFDHEKNQPSERDFRNFINEMDDGAESDSSSDLFELQNYDLGCCTYSSGLPVYETTHMSSIKR
DcFInDel14_alt_    --DHEKNQPSERDFRNFINEMDDGAESDSSSDLFELQNYDLGCCTYSSGLPVYETTHMSSIKR
cons                 *************************************************************

DcFInDel14_ref_    GAPMITS
DcFInDel14_alt_    GAPMITS
cons               *******
```

d) DcFInDel19 - XM_017400085.2 (NAC domain-containing protein 41)

```
DcFInDel19_ref_    MEKNCIVGRNGEELQLPVGFRFRPTDEELILYYLMPKAQSLPLPAAFIPQIDEIFQSHPSHLP
DcFInDel19_alt_    MEKNCIVGRNGEELQLPVGFRFRPTDEELILYYLMPKAQSLPLPAAFIPQIDEIFQSHPSHLP
cons               ***************************************************************

DcFInDel19_ref_    GDVEQRRYYFCKRSWDYSKTCRSRINYISNESSYWKQAGKERAISVDVAHRRSIVVGTKKLFV
DcFInDel19_alt_    GDVEQRRYYFCKRSWDYSKTCRSRINYISNESSYWKQAGKERAISVDVAHRRSIVVGTKKLFV
cons               ***************************************************************

DcFInDel19_ref_    LYEEKQKTSWCMQEYRLLPSQFEDFD-----NWVAYRVYQRKRNGRVKNTRENTKKMDVAEGV
DcFInDel19_alt_    LYEEKQKTSWCMQEYRLLPSQFPAISTEDFDNWVAYRVYQRKRNGRVKNTRENTKKMDVAEGV
cons               **********************  :.     ********************************

DcFInDel19_ref_    EMMSINNATEVESSPLPSPLCSSNDEETFL
DcFInDel19_alt_    EMMSINNATEVESSPLPSPLCSSNDEETFL
cons               ******************************
```

e) DcFInDel20 - XM_017363643.2 (BURP domain protein RD22)

```
DcFInDel20_ref_    MEFKVVHFVAILSVAFVASHAAVSCEDYWRSVLPNTPMPKSISELLRSPEWMDDKSTAVDVGE
DcFInDel20_alt_    MEFKVVHFVAILSVAFVASHAAVSCEDYWRSVLPNTPMPKSISELLRSPEWMDDKSTAVDVGE
cons               ***************************************************************

DcFInDel20_ref_    GNVGVETGTTGGPGTNVQVGKGTGVGVSTGAPGDETDVGVGKGGVVVRSDHKGKPVYVGVSPG
DcFInDel20_alt_    GNVGVETGTTGGPGTN----------------GDETDVGVGKGGVVVRSDHKGKPVYVGVSPG
cons               ****************                *******************************

DcFInDel20_ref_    SNPFIYNYAASAAQLHDDPNVALFFLEKDLHQGANMELHFTRPTTQTPFLPRYVADSIPFSSN
DcFInDel20_alt_    SNPFIYNYAASAAQLHDDPNVALFFLEKDLHQGANMELHFTRPTTQTPFLPRYVADSIPFSSN
cons               ***************************************************************

DcFInDel20_ref_    KVLEIFEKFSVKQNTLESEAIKNTLKECEAPGIKGEEKYCATSLENMIDFTASKLGKKVSAVS
DcFInDel20_alt_    KVLEIFEKFSVKQNTLESEAIKNTLKECEAPGIKGEEKYCATSLENMIDFTASKLGKKVSAVS
cons               ***************************************************************

DcFInDel20_ref_    TVVEKESEMQRFSIIGSKKLGEKAVICHKQSYPYAVFYCHETNNVKAYTVSLVGNDGTKAKAA
DcFInDel20_alt_    TVVEKESEMQRFSIIGSKKLGEKAVICHKQSYPYAVFYCHETNNVKAYTVSLVGNDGTKAKAA
cons               ***************************************************************

DcFInDel20_ref_    AICHTDTSSWNPKHLAFQVLNVKPGSVPVCHFLPEDHVVWVPY
DcFInDel20_alt_    AICHTDTSSWNPKHLAFQVLNVKPGSVPVCHFLPEDHVVWVPY
cons               *******************************************
```

f) DcFInDel21 - XM_064090015.1 (ubiquitin carboxyl-terminal hydrolase 18)

```
DcFInDel21_ref_    MLLRLILSFLRLPHSDLNLFILQFLFTVCFALALLSFVKHTASNYFVIDDNFAPKMSEFVQCE
DcFInDel21_alt_    MLLRLILSFLRLPHSDLNLFILQFLFTVCFALALLSFVKHTASNYFVIDDNFAPKMSEFVQCE
cons               ************************************************************

DcFInDel21_ref_    VCGQASTKKCSACKLVRYCSEACQKSHWNSEHKKTCKSFQLSNKANLKPSGSGVQLKIPSTAR
DcFInDel21_alt_    VCGQASTKKCSACKLVRYCSEACQKSHWNSEHKKTCKSFQLSNKANLKPSGSGVQLKIPSTAR
cons               ************************************************************

DcFInDel21_ref_    RQSFKNLKDPNQVLFSYDEFVQLFKRERTGTRPCGLLNCGNSCFANVVLQCLACTRPLVAYLL
DcFInDel21_alt_    RQSFKNLKDPNQVLFSYDEFVQLFKRERTGTRPCGLLNCGNSCFANVVLQCLACTRPLVAYLL
cons               ************************************************************

DcFInDel21_ref_    EKDHRRECWRNDWCFMCELQSHVTRATQTSQPFSPLEILARLPNIGGNLGYGKQEDAHEFMRF
DcFInDel21_alt_    EKDHRRECWRNDWCFMCELQSHVTRATQTSQPFSPLEILARLPNIGGNLGYGKQEDAHEFMRF
cons               ************************************************************

DcFInDel21_ref_    AIDTMQSVCLDEFGGEKVVPPRAQETTLIQHIFGGQLQSQVKCTKCENVSNQFENMMDLTVEM
DcFInDel21_alt_    AIDTMQSVCLDEFGGEKVVPPRAQETTLIQHIFGGQLQSQVKCTKCENVSNQFENMMDLTVEM
cons               ************************************************************

DcFInDel21_ref_    QGDATSLEECLDQFTIMERLHGDNMYKCDGCNDYVLAWKRLTIRRAPNILTIALKRFQSGRFG
DcFInDel21_alt_    QGDATSLEECLDQFTIMERLHGDNMYKCDGCNDYVLAWKRLTIRRAPNILTIALKRFQSGRFG
cons               ************************************************************

DcFInDel21_ref_    KLNKRVSFPETLDLSPYMSEADDDGNVYKLYAVVVHVDMLNASYFGHYICYTKDFSGNWYRID
DcFInDel21_alt_    KLNKRVSFPETLDLSPYMSEADDDGNVYKLYAVVVHVDMLNASYFGHYICYTKDFSGNWYRID
cons               ************************************************************

DcFInDel21_ref_    DCKVYKVEMDEVLSQGAYMLLYSRVSARASCLNPIEPASKHGGEKLEVALEVGPSAKQAVEEF
DcFInDel21_alt_    DCKVYKVEMDEVLSQGAYMLLYSRVSARASCLNPIEPASKHGGEKLEVALEVGPSAKQAVEEF
cons               ************************************************************

DcFInDel21_ref_    SAVDSIDSPIVSGPCLSVTNSQEMNSGCEDAKDLEMVDSEASSSALTDLDIHGSTCCNEAEGS
DcFInDel21_alt_    SAVDSIDSPIVSGPCLSVTNSQEMNSGCEDAKDLEMVDSEASSSALTDLDIHGSTCCNEAEGS
cons               ************************************************************

DcFInDel21_ref_    LSDLEMSQGQISEVSSSIITQPNNEPEQPFPVFEVENKDMAVESRGKMHALINPLDNDIEPCS
DcFInDel21_alt_    LSDLEMSQGQISEVSSSIITQPNNEPEQPFPVFEVENKDMAVESRGKMHALINPLDNDIEPCS
cons               ************************************************************

DcFInDel21_ref_    NGVCGNGDHADAGATLNTGEVLNNTCLCSSTGKVEKVKESGKDMPSSQITAGAGKYNGRNSSG
DcFInDel21_alt_    NGVCGNGDHADAGATLNTGEVLNNTCLCSSTGKVEKVKESGKDMPSSQITAGAGKYNGRNSSG
cons               ************************************************************

DcFInDel21_ref_    LKPKPLFASGFLEKHPLNKQSKEEIKAPVQIGQLASVCNYNGKKNSDPDLSSKNENGTGGGGP
DcFInDel21_alt_    LKPKPLFASGFLEKHPLNKQSKEEIKAPVQIGQLASVCNYNGKKNSDPDLSSKNENGTGGGGP
cons               ************************************************************

DcFInDel21_ref_    HILSGIPRKQTAKSEDGATSKVTDSHTPAMRSSDSIDVPLDSLDNGNSSSSGEVSIPVSNGVT
DcFInDel21_alt_    HILSGIPRKQTAKSEDGATSKVTDSHTPAMRSSDSIDVPLDSLDNGNSSSSGEVSIPVSNGVT
cons               ************************************************************

DcFInDel21_ref_    FNSTTAGVLTELHFSFSNSDGKPKKLEINKDSLALPVNKDVSAGGKDGRKHSGSESKPLLRPG
DcFInDel21_alt_    FNSTTASFSNSD--------GKPKKLEINKDSLALPVNKDVSAGGKDGRKHSGSESKPLLRPG
cons               ******.. ..         ****************************************

DcFInDel21_ref_    FLGKHPREKYSKQEAVVPAEIGNASSNSACKLNGTSSNRYVLPGISREYMGDSEDGDSYVGNS
DcFInDel21_alt_    FLGKHPREKYSKQEAVVPAEIGNASSNSACKLNGTSSNRYVLPGISREYMGDSEDGDSYVGNS
cons               ************************************************************

DcFInDel21_ref_    SSSDI
DcFInDel21_alt_    SSSDI
cons               *****
```

g) DcFInDel22 - XM_017362733.2 (protein TILLER ANGLE CONTROL)

```
DcFInDel22_ref_    MKIFNWVHRRFHNKDESSENVKMVKAEHGTGALLEQVDVIQDWGQGILAIGTFGYDPSPNDFN
DcFInDel22_alt_    MKIFNWVHRRFHNKDESSENVKMVKAEHGTGALLEQVDVIQDWGQGILAIGTFGYDPSPNDFN
cons               ************************************************************

DcFInDel22_ref_    QQVQMFHMFENEDELELLEEEGEKEDGVEGENKICDHDQGRELNPLVLKASKHGFSNEDNVCD
DcFInDel22_alt_    QQVQMFHMFENEDELELLEEEGEKEDGVEGENKICDHDQGRELNPLVLK---------DNVCD
cons               ************************************************          *****

DcFInDel22_ref_    HDQNAIKSEITILSVDDVENSECVVREVYEKKERTTLADLFMADSDEYKDQGEVVNKPKSIKK
DcFInDel22_alt_    HDQNAIKSEITILSVDDVENSECVVREVYEKKERTTLADLFMADSDEYKDQGEVVNKPKSIKK
cons               ************************************************************

DcFInDel22_ref_    AEGAENSKFKLPFAKKFIPLMKQDNSCSSSHPCGIKKIHKMMRRMLKKKVHPELESKRQKPFK
DcFInDel22_alt_    AEGAENSKFKLPFAKKFIPLMKQDNSCSSSHPCGIKKIHKMMRRMLKKKVHPELESKRQKPFK
cons               ************************************************************

DcFInDel22_ref_    YGGNEVMEMASLLQTQDAIICP
DcFInDel22_alt_    YGGNEVMEMASLLQTQDAIICP
cons               **********************
```

h) DcFInDel26 - XM_064088842.1 (cytochrome P450 734A1-like isoform X1)

```
DcFInDel26_ref_    MILLVLLLVIILSKLTHKHIWIPLRIQRHFRNQGIKGPAYRIFLGNSAEIRRLMMAEAESTAM
DcFInDel26_alt_    MILLVLLLVIILSKLTHKHIWIPLRIQRHFRNQGIKGPAYRIFLGNSAEIRRLMMAEAESTAM
cons               ************************************************************

DcFInDel26_ref_    AFNHDIVQRVMPHYYNWSKEYGKNFLYWFGPKPRLAVADPDLIKEVLLDSSGCFVKPKLNPSA
DcFInDel26_alt_    AFNHDIVQRVMPHYYNWSKEYGKNFLYWFGPKPRLAVADPDLIKEVLLDSSGCFVKPKLNPSA
cons               ************************************************************

DcFInDel26_ref_    KLLFGEGLVGLSGEKWGVHRRITAQAFNMERVKDWVPEMVASTRKMLDRWEEESGGRDKYEID
DcFInDel26_alt_    KLLFGEGLVGLSGEKWGVHRRITAQAFNMERVKDWVPEMVASTRKMLDRWEEESGGRDKYEID
cons               ************************************************************

DcFInDel26_ref_    VHKELHKLSADIISRTAFGSNFEEGKHVFELQDQQTGLVLQALRSVYVPGFKFLPTKKNRMRW
DcFInDel26_alt_    VHKELHKLSADIISRTAFGSNFEEGKHVFELQDQQTGLVLQALRSVYVPGFKFLPTKKNRMRW
cons               ************************************************************

DcFInDel26_ref_    RLEKETQQSIRTILENKKSSDSPKCLLTLLMSPYKNQENKEEKLSSEEITDECKTFYFAGKET
DcFInDel26_alt_    RLEKETQQSIRTILENKKSSDSPKCLLTLLMSPYKNQENKEEKLSSEEITDECKTFYFAGKET
cons               ************************************************************

DcFInDel26_ref_    TANHLTWALLLLALHQEWQEKAREEVSRVCGQSSSPSADNSADLKIINMILNETLRLYPPAVM
DcFInDel26_alt_    TANHLTWALLLLALHQEWQEKARDRVHLQVLIIQPT---------------------------
cons               ***********************:.*       ...

DcFInDel26_ref_    LMRETNRTVKLGSLEIPAETQLYLPMTAIHHDTEIWGADAKEFNPMRFGESRKHLAAFFPFSL
DcFInDel26_alt_    ------------------------------------------------------------
cons

DcFInDel26_ref_    GSRICVGQNLAMVEAKIVLAMVIQRYYLEISPSYVHAPRQLMTMQPQFGAQILFTRIP
DcFInDel26_alt_    ----------------------------------------------------------
cons
```

i) DcFInDel37 - XM_017394094.2 (probable calcium-binding protein CML36)

```
DcFInDel37_ref_    MPLNHPPFKPPLFPLHFFITPHFSLISPLSSISLYIIYPAMKLSNINPKKLFKKKTRSVSRSE
DcFInDel37_alt_    MPLNHPPFKPPLFPLHFFITPHFSLISPLSSISLYIIYPAMKLSNINPKKLFKKKTRSVSRSE
cons               ************************************************************

DcFInDel37_ref_    QSSFGSSATSSSDDSTHHSKPAGSGTPTSVLPDPTSSDFCDLVQAFKIIDADGDGKITRAELG
DcFInDel37_alt_    QSSFGSSATSSSDDSTHHSKPAGSGTPTSVLPDPTSSDFCDLVQAFKIIDADGDGKITRAELG
cons               ************************************************************

DcFInDel37_ref_    SLLGRVGSDPLSEEELTMMLRELDSDGDGCISLEEFGAISSAFGPPSGDVELREAFDFFDTDH
DcFInDel37_alt_    SLLGRVGSDPLSEEELTMMLRELDSDGDGCISLEEFGAISSAFGPPSGDVELREAFDFFDTDH
cons               ************************************************************
DcFInDel37_ref_    DGKISAEELLCVFTSIGDGVCSLEDCRRMIKGVDKNMDGFVCFEDFSKMMLHQYR
```

```
DcFInDel37_alt_    DGKISAEELLCVFTSIGDGVCSLEDCRRMIKGVDKNMDGFVCFEDFSKMMLHQYR
cons               *******************************************************
```

j) DcFInDel43 - XM_017363925.2 (zinc finger protein BRUTUS-like At1g74770)

```
DcFInDel43_ref_    MFRNEDSGCRAGEVAAVHQNGVGLVDNPILVLVYFHKALRAEFAELRRVAVEALESGTHGVEL
DcFInDel43_alt_    MFRNEDSGCRAGEV-------VGLVDNPILVLVYFHKALRAEFAELRRVAVEALESGTHGVEL
cons               **************       ***** ************************************

DcFInDel43_ref_    LVMLRKRFEFLKLFYKYHSAAEDEVIFLALDELVKNVVSTYSLEHKSIDDLFDSVFNCLDVLD
DcFInDel43_alt_    LVMLRKRFEFLKLFYKYHSAAEDEVIFLALDELVKNVVSTYSLEHKSIDDLFDSVFNCLDVLD
cons               ***************************************************************

DcFInDel43_ref_    KEGKDNYKTFQGLLFCIGTIQTTIHQHMLKEEEQVFPLLMQQFSSSEQASLVWQFMCSVPVTL
DcFInDel43_alt_    KEGKDNYKTFQGLLFCIGTIQTTIHQHMLKEEEQVFPLLMQQFSSSEQASLVWQFMCSVPVTL
cons               ***************************************************************

DcFInDel43_ref_    LEDFLPWMISVLSPDEKIEVSRSMKEIVPKEKLLQEVVISWIDNEVPATGGCTSIREQGAQFN
DcFInDel43_alt_    LEDFLPWMISVLSPDEKIEVSRSMKEIVPKEKLLQEVVISWIDNEVPATGGCTSIREQGAQFN
cons               ***************************************************************

DcFInDel43_ref_    NGLGTHRETSIQHSFDGIRLWHDAIRKDLQEVLIELYQIRISSEFSDLPSLVVDLNFLADTLI
DcFInDel43_alt_    NGLGTHRETSIQHSFDGIRLWHDAIRKDLQEVLIELYQIRISSEFSDLPSLVVDLNFLADTLI
cons               ***************************************************************

DcFInDel43_ref_    FYSKALSNIIYPLWNELAKDFYSARYAQYLDERKIEGLQRLLYYKSEKTIPLRSYVEKLYEEL
DcFInDel43_alt_    FYSKALSNIIYPLWNELAKDFYSARYAQYLDERKIEGLQRLLYYKSEKTIPLRSYVEKLYEEL
cons               ***************************************************************

DcFInDel43_ref_    MSFASWIDENLSLIEAEVFPLIRMNCSRDMQQWLLYTCLKMMPLGLIKCVITWFSAHLSGNEA
DcFInDel43_alt_    MSFASWIDENLSLIEAEVFPLIRMNCSRDMQQWLLYTCLKMMPLGLIKCVITWFSAHLSGNEA
cons               ***************************************************************

DcFInDel43_ref_    KSALKINLEGPLARNPLASLLCHWLRISYSGKSSIEKFKEDLREMFNSRCLLLCKQIKEDSEL
DcFInDel43_alt_    KSALKINLEGPLARNPLASLLCHWLRISYSGKSSIEKFKEDLREMFNSRCLLLCKQIKEDSEL
cons               ***************************************************************

DcFInDel43_ref_    SHLPLDDKHHNTSSCRKPQKSTDTENKFPTFSCSSTKRNRKCDTSYTSGMNFHVSFPQILNIP
DcFInDel43_alt_    SHLPLDDKHHNTSSCRKPQKSTDTENKFPTFSCSSTKRNRKCDTSYTSGMNFHVSFPQILNIP
cons               ***************************************************************

DcFInDel43_ref_    SYLSQNPEVSSIVSSFTILDTNPMDHIIYFHKALKKDLEYLVLVSAKLAENVGFLMDFHRCFH
DcFInDel43_alt_    SYLSQNPEVSSIVSSFTILDTNPMDHIIYFHKALKKDLEYLVLVSAKLAENVGFLMDFHRCFH
cons               ***************************************************************

DcFInDel43_ref_    LLQFFYQIHSDSEDNIAFPALEAKGNFQNISHSYSIDHKLEGEQFIKVSNILDEISKLRTYSD
DcFInDel43_alt_    LLQFFYQIHSDSEDNIAFPALEAKGNFQNISHSYSIDHKLEGEQFIKVSNILDEISKLRTYSD
cons               ***************************************************************

DcFInDel43_ref_    ADVDASGHQQPKYGQLCFKLHAMCISMHKVLCDHIDHEEIELLPLYREYFSVEEQMKITGNML
DcFInDel43_alt_    ADVDASGHQQPKYGQLCFKLHAMCISMHKVLCDHIDHEEIELLPLYREYFSVEEQMKITGNML
cons               ***************************************************************

DcFInDel43_ref_    GRMRAESLQELIPWLVASLTPEEQQAMMSLWRKATKNTKFDEWLGEWWEGGLFTAVEKVEEKQ
DcFInDel43_alt_    GRMRAESLQELIPWLVASLTPEEQQAMMSLWRKATKNTKFDEWLGEWWEGGLFTAVEKVEEKQ
cons               ***************************************************************

DcFInDel43_ref_    TNTLPTYTGDAVEVVLKYMVEEGAHDNGGIIHDRSTGISQNEISGCKRDPCGVPSATEENHRL
DcFInDel43_alt_    TNTLPTYTGDAVEVVLKYMVEEGAHDNGGIIHDRSTGISQNEISGCKRDPCGVPSATEENHRL
cons               ***************************************************************

DcFInDel43_ref_    KEDHCKDNYTVEADKVRGKEITGNSDLDETGKLVQASQHLKEERDLPILSQEELVAAIRRVHN
DcFInDel43_alt_    KEDHCKDNYTVEADKVRGKEITGNSDLDETGKLVQASQHLKEERDLPILSQEELVAAIRRVHN
cons               ***************************************************************

DcFInDel43_ref_    DSKLDLASKARIIQSLHTSRSSAAQPKVNSETTRSRNEENVSGQSASYRDPLKLTFGCKHYKR
DcFInDel43_alt_    DSKLDLASKARIIQSLHTSRSSAAQPKVNSETTRSRNEENVSGQSASYRDPLKLTFGCKHYKR
cons               ***************************************************************
```

```
DcFInDel43_ref_     NCKLVSACCNKLYTCRLCHDDVEDHIMDRKDTAKMMCMKCLIIQPVGATCSTPSCQKLSMAKY
DcFInDel43_alt_     NCKLVSACCNKLYTCRLCHDDVEDHIMDRKDTAKMMCMKCLIIQPVGATCSTPSCQKLSMAKY
cons                ***************************************************************

DcFInDel43_ref_     YCSICKFFDDEREIYHCPYCNLCRLGKGLGIDYFHCMNCNACMSRSLSVHICREKCFEDFCPI
DcFInDel43_alt_     YCSICKFFDDEREIYHCPYCNLCRLGKGLGIDYFHCMNCNACMSRSLSVHICREKCFEDFCPI
cons                ***************************************************************

DcFInDel43_ref_     CHEFIFSSSLPVKALQCGHLMHSSCFQAYTCSYYTCPICSKSLGDMQVYFGMLDALLAEEKIP
DcFInDel43_alt_     CHEFIFSSSLPVKALQCGHLMHSSCFQAYTCSYYTCPICSKSLGDMQVYFGMLDALLAEEKIP
cons                ***************************************************************

DcFInDel43_ref_     IEHAGQTQVILCNDCEKRGDSPFHWLYHKCPHCGSFNTRVV
DcFInDel43_alt_     IEHAGQTQVILCNDCEKRGDSPFHWLYHKCPHCGSFNTRVV
cons                *****************************************
```

k) DcFInDel44 - XM_017360436.2 (dirigent protein 22)

```
DcFInDel44_ref_     MASLFQKNHASHLITFSILISFLTFPTTGENH------DRKTLEIGSEKMSHFRVYWHDTVTG
DcFInDel44_alt_     MASLFQKNHASHLITFSILISFLTFPTTGENHDLLKSHDRKTLEIGSEKMSHFRVYWHDTVTG
cons                ********************************      *************************

DcFInDel44_ref_     PAPTAVTIVKPYYNNSSTEFGLVRMIDNPLTEGPDVKSKLIGRAQGFYGSAAQENISLLMSMN
DcFInDel44_alt_     PAPTAVTIVKPYYNNSSTEFGLVRMIDNPLTEGPDVKSKLIGRAQGFYGSAAQENISLLMSMN
cons                ***************************************************************

DcFInDel44_ref_     FAFLEGKYNGSTITVFGRNEVFNKVREMPVIGGSGLFRFARGYVQASTYSFDLKSGNAVVLYD
DcFInDel44_alt_     FAFLEGKYNGSTITVFGRNEVFNKVREMPVIGGSGLFRFARGYVQASTYSFDLKSGNAVVLYD
cons                ***************************************************************

DcFInDel44_ref_     VYVMHY
DcFInDel44_alt_     VYVMHY
cons                ******
```

l) DcFInDel45 - XM_017362876.2 (protein ESSENTIAL FOR POTEXVIRUS ACCUMULATION 1 isoform X2)

```
DcFInDel45_ref_     MAERKLNLPDDLLSSKSADQSWIPHVEASGGNADEKMLGMLDESKDLLVSESSIPLSPQWLYA
DcFInDel45_alt_     MAERKLNLPDDLLSSKSADQSWIPHVEASGGNADEKMLGMLDESKDLLVSESSIPLSPQWLYA
cons                ***************************************************************

DcFInDel45_ref_     KPSDTKMEMRAPSTLSLGNSADANQKEAWRPEGADEKKDWRRIPTDADSGRRWREEERETGLL
DcFInDel45_alt_     KPSDTKMEMRAPSTLSLGNSADANQKEAWRPEGADEKKDWRRIPTDADSGRRWREEERETGLL
cons                ***************************************************************

DcFInDel45_ref_     GRRDRRKTDRRVENAPGRESPDNRTVPAADRWHDVNNRGASHEAKRDGKWSSRWGPDDKEKEA
DcFInDel45_alt_     GRRDRRKTDRRVENAPGRESPDNRTVPAADRWHDVNNRGASHEAKRDGKWSSRWGPDDKEKEA
cons                ***************************************************************

DcFInDel45_ref_     RTEKRADAEKEDVHGDNQMNVNNNRAVSERDADARDKWRPRHRMEGNSSGPGSYRAAPGFGPE
DcFInDel45_alt_     RTEKRADAEKEDVHGDNQMNVNNNRAVSERDADARDKWRPRHRMEGNSSGPGSYRAAPGFGPE
cons                ***************************************************************

DcFInDel45_ref_     RGRVEGSNVGFAVGRGRASAVVRPPSSGPIGDADFDKERSVPGKLSFSREAYCYPRAKLLDIY
DcFInDel45_alt_     RGRVEGSNVGFAVGRGRASAVVRPPSSGPIGDADFDKERSVPGKLSFSREAYCYPRAKLLDIY
cons                ***************************************************************

DcFInDel45_ref_     RRHQHDPSFATMPDSLEEIPSVTQLTTVDPLAFVAPSIEEEAIVADIWNGKVINSGSSYNAYR
DcFInDel45_alt_     RRHQHDPSFATMPDSLEEIPSVTQLTTVDPLAFVAPSIEEEAIVADIWNGKVINSGSSYNAYR
cons                ***************************************************************

DcFInDel45_ref_     KAKSSENSGDVVDLEDTTSKQGSLPVSISEMLVDSSERYQDDDVNQADDARFFPNEGDVHHDM
DcFInDel45_alt_     KAKSSENSGDVVDLEDTTSKQGSLPVSISEMLVDSSERYQDDDVNQADDARFFPNEGDVHHDM
cons                ***************************************************************

DcFInDel45_ref_     ARKTPMTLEGIGLDKITSKMSISNDSSRAQELSDAYQFASHMKNSDLAFAKHPLFDGIESNSS
DcFInDel45_alt_     ARKTPMTLEGIGLDKITSKMSISNDSSRAQELSDAYQFASHMKNSDLAFAKHPLFDGIESNSS
cons                ***************************************************************
```

```
DcFInDel45_ref_    LSNVTTLAVDLSSSHDVKSSEQYYFGNKQVSEERPDYSEYQLERITPPEELSLFYCDPQGEIQ
DcFInDel45_alt_    LSNVTTLAVDLSSSHDVKSSEQYYFGNKQVSEERPDYSEYQLERITPPEELSLFYCDPQGEIQ
cons               ***************************************************************

DcFInDel45_ref_    GPFLGVDIISWFEQGFFGADLPVRLADASEETPFRQLGDVMPHLQGMTEYASNPDRSSKVETS
DcFInDel45_alt_    GPFLGVDIISWFEQGFFGADLPVRLADASEETPFRQLGDVMPHLQGMTEYASNPDRSSKVETS
cons               ***************************************************************

DcFInDel45_ref_    GAFEGMLDAKLSASLPVPEMMDNPRWQMSDFDGISANNVQSRMSEHEGLLDVPYSEGQSFQEF
DcFInDel45_alt_    GAFEGMLDAKLSASLPVPEMMDNPRWQMSDFDGISANNVQSRMSEHEGLLDVPYSEGQSFQEF
cons               ***************************************************************

DcFInDel45_ref_    VAQDEEIVFPGRPGSSGNPGRTSRGAGDLPASMFGEPSAKELLDSRMQSSKSNQLHPFGLLWS
DcFInDel45_alt_    VAQDEEIVFPGRPGSSGNPGRTSRGAGDLPASMFGEPSAKELLDSRMQSSKSNQLHPFGLLWS
cons               ***************************************************************

DcFInDel45_ref_    ELEGTYSRNDNNSNMPFNGGIQDQHMNSIGLRGASLASADSTHGADSWPDAYRSNMPSEANMY
DcFInDel45_alt_    ELEGTYSRNDNNSNMPFNGGIQDQHMNSIGLRGASLASADSTHGADSWPDAYRSNMPSEANMY
cons               ***************************************************************

DcFInDel45_ref_    HDVMDDIQLSRFDQESKRFDLAENLLPQQFQQHHLQQHNMLSHGHLNDPILERVSNRNMMHQQ
DcFInDel45_alt_    HDVMDDIQLSRFDQESKRFDLAENLLPQQFQQHHLQQHNMLSHGHLNDPILERVSNRNMMHQQ
cons               ***************************************************************

DcFInDel45_ref_    QLASQTGQDLEHLLALQLQQQRQLQLQQHHQLQQQQQQQILLKEQQQTQARHQLLEQLLQNQM
DcFInDel45_alt_    QLASQTGQDLEHLLALQLQQQRQLQLQQHHQLQQQQQQQILLKEQQQTQARHQLLEQLLQNQM
cons               ***************************************************************

DcFInDel45_ref_    GEPVRGQSRLDAVRSINAVDQVLLNQHMVNELQRPHHLSSNVDPSIEHLIQAKFGQTMHHGHQ
DcFInDel45_alt_    GEPVRGQSRLDAVRSINAVDQVLLNQHMVNELQRPHHLSSNVDPSIEHLIQAKFGQTMHHGHQ
cons               ***************************************************************

DcFInDel45_ref_    QSDLMDLISRSKHGQMQPMEHQILQEQLNGRQLAMGLRQRVEMEERQRGSAWPVDESIQFLGN
DcFInDel45_alt_    QSDLMDLISRSKHGQMQPMEHQILQEQLNGRQLAMGLRQRVEMEERQRGSAWPVDESIQFLGN
cons               ***************************************************************

DcFInDel45_ref_    SGGGAHRSGSAGIGQLEFYQQQQRPSPEEHMSNLERNLSLQDRIQRGLYNPSLMQFERSMSLP
DcFInDel45_alt_    SGGGAHRSGSAGIGQLEFYQQQQRPSPEEHMSNLERNLSLQDRIQRGLYNPSLMQFERSMSLP
cons               ***************************************************************

DcFInDel45_ref_    GGGPGMSLDMINSMARGQGLGMQEPSARMQPAGQGPGFSGVYSHQSSHPSSANLFHPSHLDAV
DcFInDel45_alt_    GGGPGMSLDMINSMARGQGLGMQEPSARMQPAGQGPGFSGVYSHQSSHPSSANLFHPSHLDAV
cons               ***************************************************************

DcFInDel45_ref_    EGQWSENNGQPPSDWMDSRIQQLQINERHKRQSEAGRAAEDPSLWMSAGSSDDTSKRLLMELL
DcFInDel45_alt_    EGQWSENNGQPPSDWMDSRIQQLQINERHKRQSEAGRAAEDPSLWMSAGSSDDTSKRLLMELL
cons               ***************************************************************

DcFInDel45_ref_    QQKPGHQPTEQLDATGVSYDKRLPPSSHSNH------SFNLLSDREPDLNQPFAMGSYGSNSG
DcFInDel45_alt_    QQKPGHQPTEQLDATGVSYDKRLPPSSHYTGTSPSNHSFNLLSDREPDLNQPFAMGSYGSNSG
cons               **************************** .       **************************

DcFInDel45_ref_    GPLHNKVIEEQVGLETTERFLLRSNSGALNDRAQYFSGMNENSQAIYPNANMTGKSSTDFLDL
DcFInDel45_alt_    GPLHNKVIEEQVGLETTERFLLRSNSGALNDRAQYFSGMNENSQAIYPNANMTGKSSTDFLDL
cons               ***************************************************************

DcFInDel45_ref_    ERKMHGSKSEVGTRKISASESSDEFVQHEGVAASNRGDMPNNVMSRHTSQAGAAAGIYDNKMQ
DcFInDel45_alt_    ERKMHGSKSEVGTRKISASESSDEFVQHEGVAASNRGDMPNNVMSRHTSQAGAAAGIYDNKMQ
cons               ***************************************************************

DcFInDel45_ref_    RSSSVGEDVKDRMAAVPLKRQENVLSKRPPVSRAASSQEGLSELASETIVRGKNILGGSTLPS
DcFInDel45_alt_    RSSSVGEDVKDRMAAVPLKRQENVLSKRPPVSRAASSQEGLSELASETIVRGKNILGGSTLPS
cons               ***************************************************************

DcFInDel45_ref_    EGRREAGGNPPNQTAEILSSKKDVRYRRTSSCGDADVSETTSFSDMLKSNAKKPPQPESHAAA
DcFInDel45_alt_    EGRREAGGNPPNQTAEILSSKKDVRYRRTSSCGDADVSETTSFSDMLKSNAKKPPQPESHAAA
cons               ***************************************************************
```

```
DcFInDel45_ref_    AATESSEGGRSGKKKGKKGRQIDPALLGFKVTSNRIMMGEIQHADDL
DcFInDel45_alt_    AATESSEGGRSGKKKGKKGRQIDPALLGFKVTSNRIMMGEIQHADDL
cons               ***********************************************
```

m) DcFInDel46 - XM_017359816.2 (BTB/POZ domain-containing protein At1g67900)

```
DcFInDel46_ref_    MKFMKIGSRADTFYTTEGVRSVSSEIFSDLMVQVEGTRYRLHKFPLLSKCLHLQRLCSESPES
DcFInDel46_alt_    MKFMKIGSRADTFYTTEGVRSVSSEIFSDLMVQVEGTRYRLHKFPLLSKCLHLQRLCSESPES
cons               ****************************************************************

DcFInDel46_ref_    SSKQVLLLPDFPGGAEAFELCAKFCYGIAITISAYNIVSARCAAEYLHMTEDVEKGNLIHKLE
DcFInDel46_alt_    SSKQVLLLPDFPGGAEAFELCAKFCYGIAITISAYNIVSARCAAEYLHMTEDVEKGNLIHKLE
cons               ****************************************************************

DcFInDel46_ref_    VFLNSCVLNGWKDSIVTLQSTKAFEAWAEDLGITSRCIEAIVSKVLSNPSKVNLSHSYSRRGK
DcFInDel46_alt_    VFLNSCVLNGWKDSIVTLQSTKAFEAWAEDLGITSRCIEAIVSKVLSNPSKVNLSHSYSRRGK
cons               ****************************************************************

DcFInDel46_ref_    DDTLSCNGAENRSKNMSKSWWAEDVAELGIDFYWRTMIAIKSSGKIPSNIVGDALRIYASRWL
DcFInDel46_alt_    DDTLSCNGAENRSKNMSKSWWAEDVAELGIDFYWRTMIAIKSSGKIPSNIVGDALRIYASRWL
cons               ****************************************************************

DcFInDel46_ref_    PQISKALNNEKQDTIARESDSVAEVALKNRFFIESLINLLPVDKNAVSCSFLLKLLKAANILK
DcFInDel46_alt_    PQISKALNNEKQDTIARESDSVAEVALKNRFFIESLINLLPVDKNAVSCSFLLKLLKAANILK
cons               ****************************************************************

DcFInDel46_ref_    VSSSSMAELAKRIGMQLEEATVSDLLIPNMSNTCKEQYDVGIVIDILDHFIQGQSPQTSPSRA
DcFInDel46_alt_    VSSSSMAELAKRIGMQLEEATVSDLLIPNMSNTCKEQYDVGIVIDILDHFIQGQSPQTSPSRA
cons               ****************************************************************

DcFInDel46_ref_    RGNFHRRRSRSAENLSELQESRRSSSASHGSKLRVARLMDGYLQVVACDVNLELLKFIAIAEA
DcFInDel46_alt_    RGNFHRRRSRSAENLSELQESRRSSSASHGSKLRVARLMDGYLQVVACDVNLELLKFIAIAEA
cons               ****************************************************************

DcFInDel46_ref_    IPEFARLSHDDLYRAIDIYLKGHPELNKSGRKRLCRVLDCKKLSIEACTHAAQNELLPLRVVV
DcFInDel46_alt_    IPEFARLSHDDLYRAIDIYLKGHPELNKSGRKRLCRVLDCKKLSIEACTHAAQNELLPLRVVV
cons               ****************************************************************

DcFInDel46_ref_    QVLFFEQARAAVATGQMITLPSNIKALLAAHDDPSRLLNSLISNKTLPPDDQWSVSGLKTPNS
DcFInDel46_alt_    QVLFFEQARAAVATGQMITLPSNIKALLAAHDDPSRLLNSLISNKTLPPDDQWSVSGLKTPNS
cons               ****************************************************************

DcFInDel46_ref_    SISTLKTKPAENDGSNKNYRDGTGKSSRVKSNTSLPSRSRTMFSRLWPIHRHGSEKT
DcFInDel46_alt_    SISTLKTKPAENDGSNKNYRDGTGKSN-----TSLPSRSRTMFSRLWPIHRHGSEKT
cons               **************************.     *************************
```

n) DcFInDel51 - XM_017368157.2 (ankyrin repeat-containing protein At2g01680-like)

```
DcFInDel51_ref_    MSIEVMEKKLYNACLKGDVHMLEALTREDELILARISLSSCFNQTPLHLACMLGHFELAKSLL
DcFInDel51_alt_    MSIEVMEKKLYNACLKGDVHMLEALTREDELILARISLSSCFNQTPLHLACMLGHFELAKSLL
cons               ****************************************************************

DcFInDel51_ref_    SYKPDFATRLDAQGRSPLHLASANGYVGIVKLLLQHDGKVVRVCDEDGRTPLHLAVMNGQQEC
DcFInDel51_alt_    SYKPDFATRLDAQGRSPLHLASANGYVGIVKLLLQHDGKVVRVCDEDGRTPLHLAVMNGQQEC
cons               ****************************************************************

DcFInDel51_ref_    VGELMKVDGEEIGTALHLCVMCNRLDVLDVILKSTEQDVLNLKDEKGNTVLHSATLLRRTQII
DcFInDel51_alt_    VGELMKVDGEEIGTALHLCVMCNRLDVLDVILKSTEQDVLNLKDEKGNTVLHSATLLRRTQII
cons               ****************************************************************

DcFInDel51_ref_    KYLLMTKSEVLKVNTVNENSLTALDIVEQMPQDVKTMEIKELLVSAGTRKAQELKPADQSVQE
DcFInDel51_alt_    KYLLMTKSEVLKVNTVNENSLTALDIVEQMPQDVKTMEIKELLVSAGTRKAQELKPADQSVQE
cons               ****************************************************************

DcFInDel51_ref_    GDEAAKTGTSNSKCLKIF-------DKFTKFTIFRETREARDDALLVAASVIAAMAYTAAISP
DcFInDel51_alt_    GDEAAKTGTSNSKCLKIFDKCLKIFDKFTKFTIFRETREARDDALLVAASVIAAMAYTAAISP
cons               ******************       **************************************
```

```
DcFInDel51_ref_    PGGVASMDAKEFPPLGSPGGSPDSLKDKYFHLNPAASLLAYFNPNLSYTFWISNNISFMASLS
DcFInDel51_alt_    PGGVASMDAKEFPPLGSPGGSPDSLKDKYFHLNPAASLLAYFNPNLSYTFWISNNISFMASLS
cons               ***************************************************************

DcFInDel51_ref_    VIFLYVSGSSLKRRFFTWLIRGAMWVTLTSMTIAYVCAVSATTGATEDYNALYPLVFGLLAWG
DcFInDel51_alt_    VIFLYVSGSSLKRRFFTWLIRGAMWVTLTSMTIAYVCAVSATTGATEDYNALYPLVFGLLAWG
cons               ***************************************************************

DcFInDel51_ref_    ILVFVTFLVLVYRFQRYIIPAIKRQGASMNKNISGDSATTSKSSNSYIV
DcFInDel51_alt_    ILVFVTFLVLVYRFQRYIIPAIKRQGASMNKNISGDSATTSKSSNSYIV
cons               *************************************************
```

o) DcFInDel54 - XM_017371676.2 (benzyl alcohol O-benzoyltransferase)

```
DcFInDel54_ref_    MAAAQEPLVFTVTRRAPELIPPAKPTPFEYKLLSDIDDQGSLRFRIPVIYFYRKRND-----K
DcFInDel54_alt_    MAAAQEPLVFTVTRRAPELIPPAKPTPFEYKLLSDIDDQGSLRFRIPVIYFYRKRNDFIDADK
cons               *********************************************************     *

DcFInDel54_ref_    MDPVKVIREAISKTLVFYYPLAGRVREGAGRKLGVECTGEGVMFIEADAEVTIEELGGDAIQP
DcFInDel54_alt_    MDPVKVIREAISKTLVFYYPLAGRVREGAGRKLGVECTGEGVMFIEADAEVTIEELGGDAIQP
cons               ***************************************************************

DcFInDel54_ref_    PFPCFEELLFDVPGYSGVLDCPILLFQVTRLKCGGFIIAVRLNHTVSDAGGLAQFVNAVGEIA
DcFInDel54_alt_    PFPCFEELLFDVPGYSGVLDCPILLFQVTRLKCGGFIIAVRLNHTVSDAGGLAQFVNAVGEIA
cons               ***************************************************************

DcFInDel54_ref_    RGADSPSVPPVWQREILNARDPPRVTCTHHEYDDVPDRKGTLISVGNMVHRSFFFGPAEILVL
DcFInDel54_alt_    RGADSPSVPPVWQREILNARDPPRVTCTHHEYDDVPDRKGTLISVGNMVHRSFFFGPAEILVL
cons               ***************************************************************

DcFInDel54_ref_    RLLVPSHLIKCSTSELLTACLWRCRTRALQLDPEEEVRLLYNINARGRFNPPLPKGYYGNAFA
DcFInDel54_alt_    RLLVPSHLIKCSTSELLTACLWRCRTRALQLDPEEEVRLLYNINARGRFNPPLPKGYYGNAFA
cons               ***************************************************************

DcFInDel54_ref_    FPAAVTTAGKLCQNPIGYALELVKKIKKDFSEEYMRSVADLMVLKGRPPSTAVRIFHVSDVTR
DcFInDel54_alt_    FPAAVTTAGKLCQNPIGYALELVKKIKKDFSEEYMRSVADLMVLKGRPPSTAVRIFHVSDVTR
cons               ***************************************************************

DcFInDel54_ref_    SGLGDADFGWGKPVYGGPARGGVGNRPVAASFYVSCKNKKGENGIAVTVSLQASVMEKFAAEL
DcFInDel54_alt_    SGLGDADFGWGKPVYGGPARGGVGNRPVAASFYVSCKNKKGENGIAVTVSLQASVMEKFAAEL
cons               ***************************************************************

DcFInDel54_ref_    DGMLKNNDQLIINNTSIPTKSAL
DcFInDel54_alt_    DGMLKNNDQLIINNTSIPTKSAL
cons               ***********************
```
