# Supplementary figures and images for: Exploring functional InDels and genetic diversity: agro-morphometric and molecular insights into the Western and Eastern gene pools of carrot (Daucus carota L.)
Source: Front Plant Sci. 2025 Oct 22;16:1658653. doi: 10.3389/fpls.2025.1658653 (PMC12587770; doi:10.3389/fpls.2025.1658653)

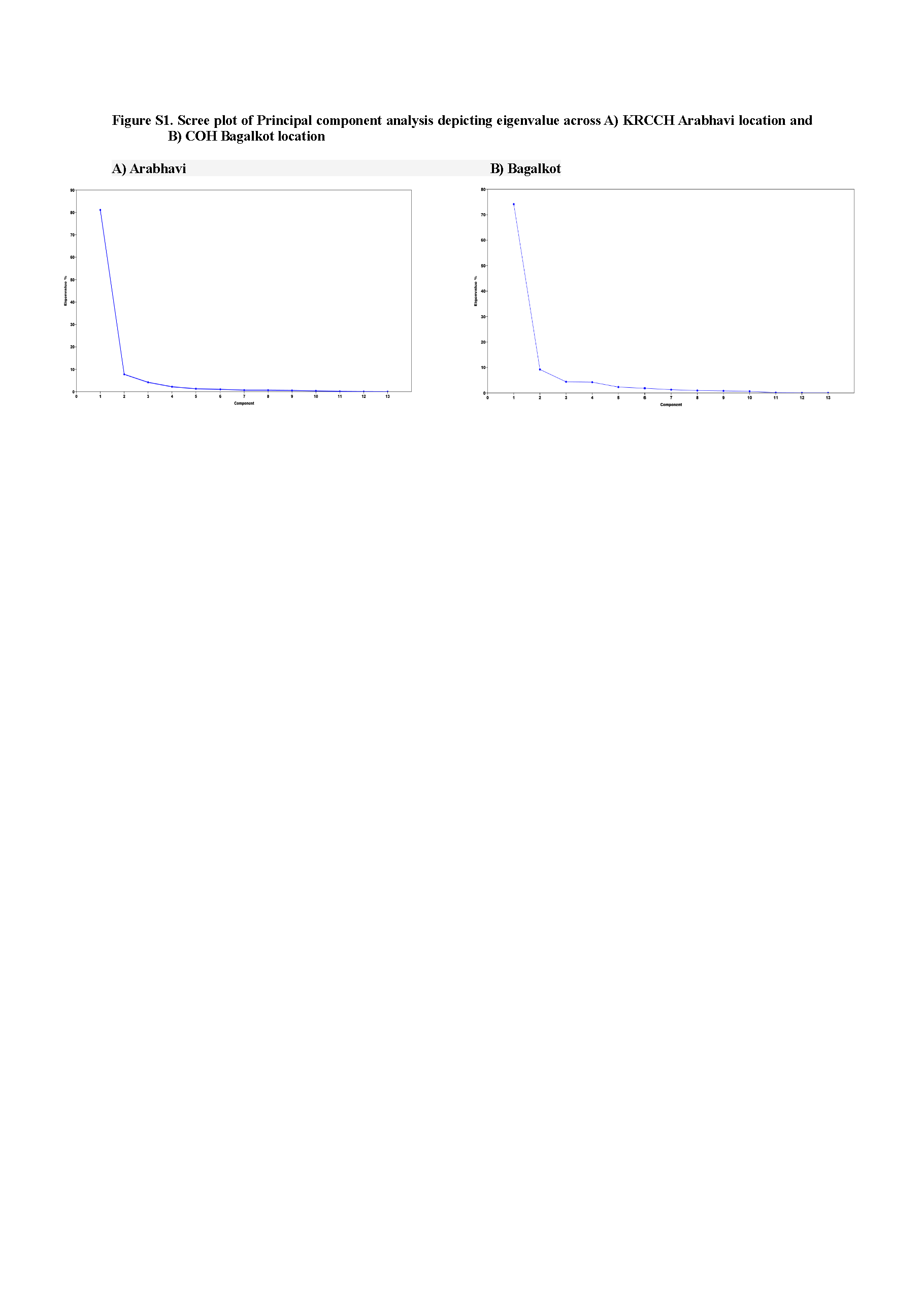

Supplement: Supplementary file 1 [file DataSheet1.zip › Supplementary Figure 1 TIFF Mudihal et al.tiff]

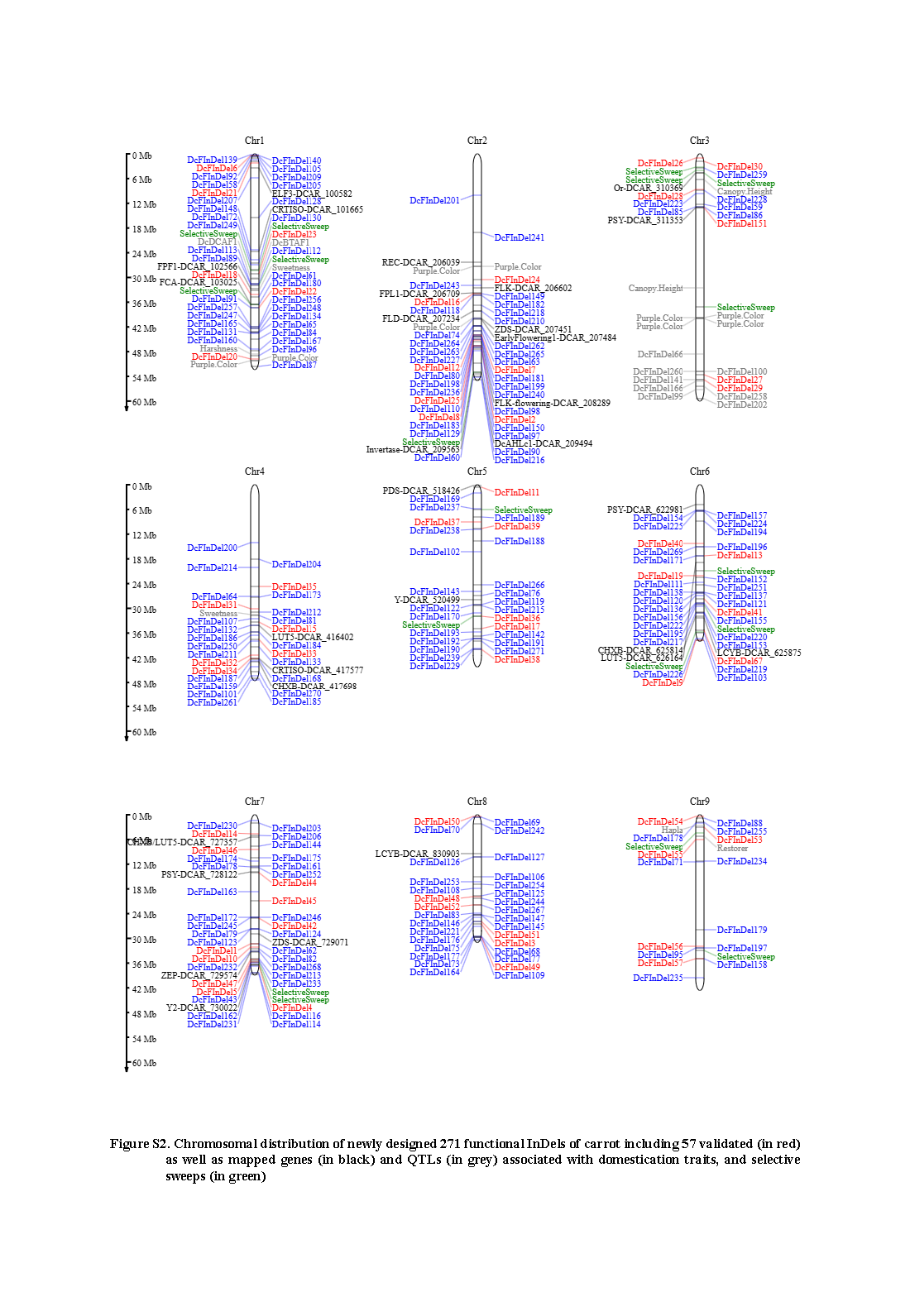

Supplement: Supplementary file 1 [file DataSheet1.zip › Supplementary Figure 2 TIFF Mudihal et al.tiff]

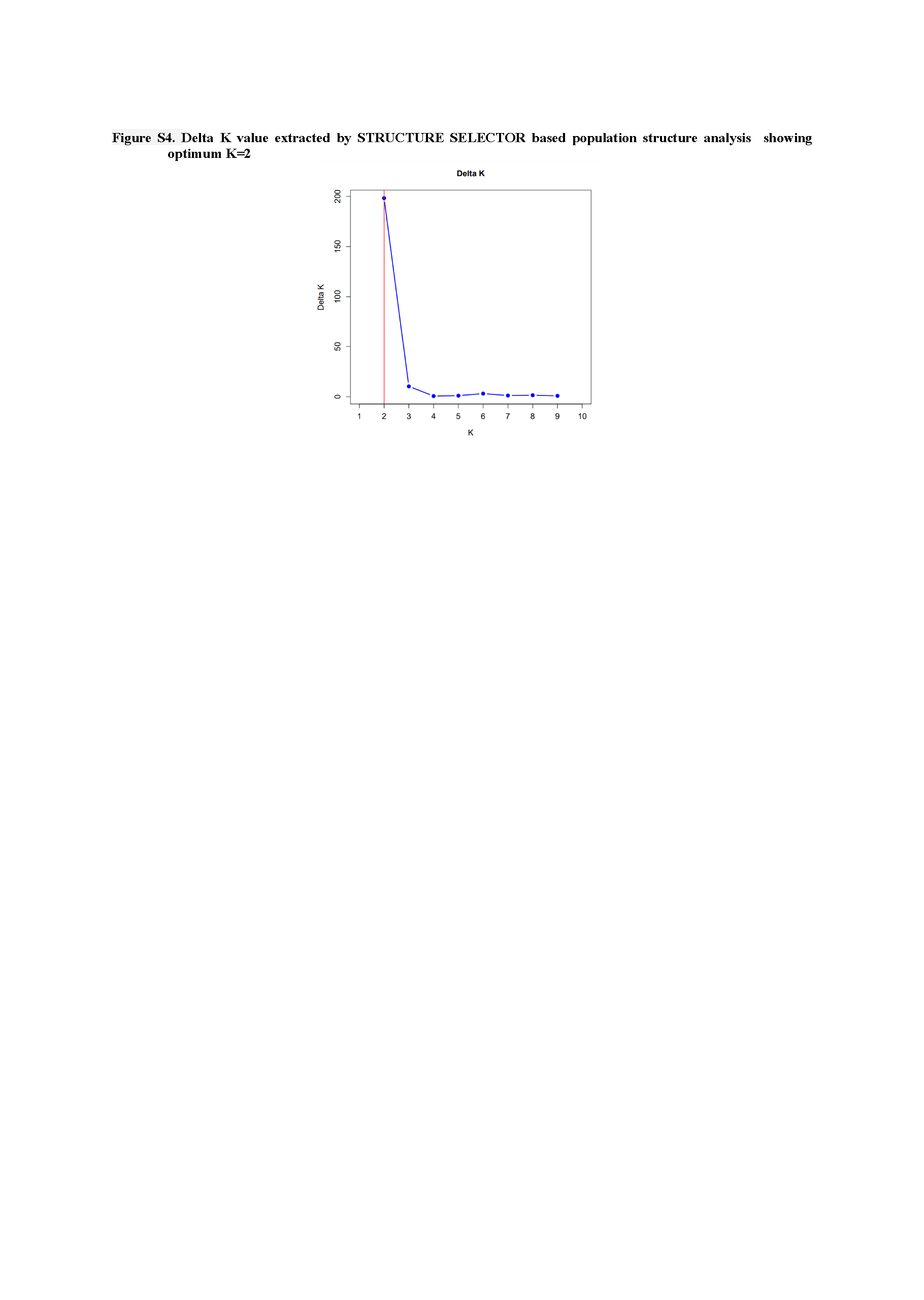

Supplement: Supplementary file 1 [file DataSheet1.zip › Supplementary Figure 4 TIFF Mudihal et al.tiff]
